# Neuropsychiatric manifestations and sleep disturbances with dolutegravir-based antiretroviral therapy versus standard of care in children and adolescents: a secondary analysis of the ODYSSEY trial

**DOI:** 10.1016/S2352-4642(23)00164-5

**Published:** 2023-08-08

**Authors:** Anna Turkova, Ellen White, Adeodata R Kekitiinwa, Vivian Mumbiro, Elizabeth Kaudha, Afaaf Liberty, Grace Miriam Ahimbisibwe, Tumelo Moloantoa, Ussanee Srirompotong, Nozibusiso Rejoice Mosia, Thanyawee Puthanakit, Robin Kobbe, Clàudia Fortuny, Hajira Kataike, Dickson Bbuye, Sathaporn Na-Rajsima, Alexandra Coelho, Abbas Lugemwa, Mutsa F Bwakura-Dangarembizi, Nigel Klein, Hilda A Mujuru, Cissy Kityo, Mark F Cotton, Rashida A Ferrand, Carlo Giaquinto, Pablo Rojo, Avy Violari, Diana M Gibb, Deborah Ford

**Affiliations:** Medical Research Council Clinical Trials Unit at University College London, London, UK; Baylor College of Medicine, Kampala, Uganda; University of Zimbabwe Clinical Research Centre, Harare, Zimbabwe; Joint Clinical Research Centre, Kampala, Uganda; Perinatal HIV Research Unit, University of the Witwarsrand, Johannesburg, South Africa; Makerere University–Johns Hopkins University Research Collaboration, Kampala, Uganda; Perinatal HIV Research Unit, University of the Witwarsrand, Johannesburg, South Africa; Pediatric Department, Khon Kaen Hospital, Thailand; Department of Paediatrics and Children Health, King Edward VIII Hospital, Enhancing Care Foundation, University of KwaZulu-Natal, Durban, South Africa; HIVNAT, Thai Red Cross AIDS Research Center, Bangkok, Thailand; Department of Pediatrics, Faculty of Medicine, Chulalongkorn University, Thailand; Institute for Infection Research and Vaccine Development, University Medical Centre Hamburg-Eppendorf, Hamburg, Germany; Department of Infectious Disease Epidemiology, Bernhard Nocht Institute for Tropical Medicine, Hamburg, Germany; Infectious Diseases Department, Institut de Recerca Sant Joan de Déu, Sant Joan de Déu Children’s Hospital, Barcelona, Spain; Department of Surgery and Medico-Surgical Specialties, Faculty of Medicine and Health Sciences, Universitat de Barcelona, Barcelona, Spain; Makerere University–Johns Hopkins University Research Collaboration, Kampala, Uganda; Baylor College of Medicine, Kampala. Uganda; Pediatric Department, Mahasarakam Hospital, Thailand; INSERM/ANRS SC10-US19, Essais Thérapeutiques et Maladies Infectieuses, Villejuif, France; Joint Clinical Research Centre, Mbarara, Uganda; University of Zimbabwe Clinical Research Centre, Harare, Zimbabwe; Infection, Immunity & Inflammation Department, UCL Great Ormond Street Institute of Child Health, London, UK; Africa Health Research Institute, Kwazulu-Natal, South Africa; University of Zimbabwe Clinical Research Centre, Harare, Zimbabwe; Joint Clinical Research Centre, Kampala, Uganda; Children’s Infectious Diseases Clinical Research Unit, Family Center for Research with Ubuntu, Department of Paediatrics and Child Health, University of Stellenbosch, Cape Town, South Africa; Department of Clinical Research, Faculty of Infectious and Tropical Diseases, London School of Hygiene and Tropical Medicine, London, UK; Department of Women and Child Health, Padova, University of Padova, Italy; Pediatric Infectious Diseases Unit, Hospital 12 de Octubre, Madrid, Spain; Perinatal HIV Research Unit, University of the Witwarsrand, Johannesburg, South Africa; Medical Research Council Clinical Trials Unit at University College London, London, UK

## Abstract

**Background:**

Cohort studies in adults with HIV showed that dolutegravir was associated with neuropsychiatric adverse events and sleep problems, yet data are scarce in children and adolescents. We aimed to evaluate neuropsychiatric manifestations in children and adolescents treated with dolutegravir-based treatment versus alternative antiretroviral therapy.

**Methods:**

This is a secondary analysis of ODYSSEY, an open-label, multicentre, randomised, non-inferiority trial, in which adolescents and children initiating first-line or second-line antiretroviral therapy were randomly assigned 1:1 to dolutegravir-based treatment or standard-of-care treatment. We assessed neuropsychiatric adverse events (reported by clinicians) and responses to the mood and sleep questionnaires (reported by the participant or their carer) in both groups. We compared the proportions of patients with neuropsychiatric adverse events (neurological, psychiatric, and total), time to first neuropsychiatric adverse event, and participant-reported responses to questionnaires capturing issues with mood, suicidal thoughts, and sleep problems.

**Findings:**

Between Sept 20, 2016, and June 22, 2018, 707 participants were enrolled, of whom 345 (49%) were female and 362 (51%) were male, and 623 (88%) were Black-African. Of 707 participants, 350 (50%) were randomly assigned to dolutegravir-based antiretroviral therapy and 357 (50%) to non-dolutegravir-based standard-of-care. 311 (44%) of 707 participants started first-line antiretroviral therapy (ODYSSEY-A; 145 [92%] of 157 participants had efavirenz-based therapy in the standard-of-care group), and 396 (56%) of 707 started second-line therapy (ODYSSEY-B; 195 [98%] of 200 had protease inhibitor-based therapy in the standard-of-care group). During follow-up (median 142 weeks, IQR 124–159), 23 participants had 31 neuropsychiatric adverse events (15 in the dolutegravir group and eight in the standard-of-care group; difference in proportion of participants with ≥1 event p=0·13). 11 participants had one or more neurological events (six and five; p=0·74) and 14 participants had one or more psychiatric events (ten and four; p=0·097). Among 14 participants with psychiatric events, eight participants in the dolutegravir group and four in standard-of-care group had suicidal ideation or behaviour. More participants in the dolutegravir group than the standard-of-care group reported symptoms of self-harm (eight *vs* one; p=0·025), life not worth living (17 *vs* five; p=0·0091), or suicidal thoughts (13 *vs* none; p=0·0006) at one or more follow-up visits. Most reports were transient. There were no differences by treatment group in low mood or feeling sad, problems concentrating, feeling worried or feeling angry or aggressive, sleep problems, or sleep quality.

**Interpretation:**

The numbers of neuropsychiatric adverse events and reported neuropsychiatric symptoms were low. However, numerically more participants had psychiatric events and reported suicidality ideation in the dolutegravir group than the standard-of-care group. These differences should be interpreted with caution in an open-label trial. Clinicians and policy makers should consider including suicidality screening of children or adolescents receiving dolutegravir.

**Funding:**

Penta Foundation, ViiV Healthcare, and UK Medical Research Council.

## Introduction

In 2021, 38·4 million people were estimated to be living with HIV, of whom 2·7 million were children and adolescents aged younger than 19 years.^1^ Mental health issues are prevalent among this population.^2^ A systematic review and meta-analysis found that about a quarter of young people living with HIV had lifetime suicidal ideations (24%, 95% CI 18–31) and one in eight made a suicide attempt (13%, 8–22).^3^ The cause of neuropsychiatric manifestations is probably multifactorial, including socioeconomic and lifestyle factors, stigma associated with HIV infection, the effect of HIV on health, and toxicities of antiretroviral therapy,^2,4^ making it challenging to assess the association with specific antiretroviral therapies in non-comparative studies.

The global scale-up of antiretroviral therapy programmes has substantially increased survival across all age groups. Dolutegravir, a second-generation anti-retroviral drug of integrase strand transfer inhibitors class, had superior efficacy compared with non-dolutegravir standard-of-care anchor drugs in adults.^5,6^ The ODYSSEY trial has also shown the superiority of dolutegravir over other standard-of-care drugs in children and adolescents starting first-line or second-line antiretroviral therapy.^7^ Dolutegravir is recommended by WHO as the preferred anchor drug for children, adolescents, and adults receiving first-line and second-line treatment,^8^ and is being rolled out globally.

Randomised controlled trials in adults have shown excellent safety of dolutegravir-based antiretroviral therapy with few toxicity-related treatment discontinuations.^6,9,10^ However, data from adult cohort studies suggest that dolutegravir is associated with neuropsychiatric manifestations and sleep problems leading to treatment discontinuation in around 4% (range 1–7%) of exposed adults.^11^ The most frequently reported dolutegravir-related adverse events are insomnia, depression, anxiety, headache, and dizziness. Suicidal ideation or behaviour are reported in fewer than one in 100 people.^12^

Data are scarce on dolutegravir-associated neuropsychiatric manifestations in children and adolescents. IMPAACT P1093, a prospective, dolutegravir dose-finding study, reported one grade 4 nervous system disorder (seizure) in their cohort of children aged 4 weeks to 6 years (n=73)^13^ and one grade 3 event of abnormal behaviour among children aged 6–12 years^14^ during 48 weeks of follow-up. Of 23 participants in the adolescent cohort (aged 12–17 years, median follow-up 153 weeks), one (4%) participant with pre-existing mental illness had grade 4 depression with a suicide attempt, four (17%) had grade 1–2 transient dizziness, and six (26%) had headaches.^15^ None of the events were considered related to dolutegravir. In the four European cohort studies reporting the use of dolutegravir in children and adolescents (aged 5–17 years), neuropsychiatric adverse events occurred infrequently, in one to three cases in each cohort (1–7% of exposed patients) over a median follow-up of 9–24 months.^16–18^

We present the secondary analysis of clinician-reported neuropsychiatric adverse events and participant or carer-completed mood and sleep questionnaires in children and adolescents enrolled in the ODYSSEY trial.^7^

## Methods

### Study design and participants

ODYSSEY was an open-label, multicentre, randomised, non-inferiority trial done in 29 centres in Africa (South Africa, Uganda, Zimbabwe), Europe (Germany, Spain, UK), and Thailand. We conducted a detailed secondary analysis of clinician-reported neuropsychiatric adverse events and sleep questionnaire responses from children (aged ≥6 years) and their carers in the main ODYSSEY trial, which enrolled children weighing 14 kg or more starting first-line or second-line therapy. This analysis did not include the cohort of patients weighing less than 14 kg who were enrolled in a separate randomised cohort in ODYSSEY (n=85, median age at enrolment 1·4 years, range 0·1 –5·9), as questionnaire data were limited, and no neuropsychiatric adverse events were reported during a median of 124 weeks of follow-up.^19^

Children or their carers (or both) gave written informed consent and assent as appropriate to participate in the ODYSSEY trial. The trial was approved by national or local ethics committees and is registered with ClinicalTrials.gov (NCT02259127).

### Randomisation and masking

Children were randomly assigned (1:1) to dolutegravir-based antiretroviral therapy or non-dolutegravir-based standard-of-care. Randomisation was stratified by ODYSSEY A (children starting first-line treatment) and ODYSSEY B (starting second-line treatment), routine availability of resistance tests, intended standard-of-care third agent, and intended nucleoside reverse transcriptase backbone. The computer-generated randomisation list was prepared by the trial statistician and incorporated within the database, enabling access only to the next allocation.

### Procedures

The efficacy and safety of dolutegravir-based treatment were compared with non-dolutegravir-based standard-of-care treatment. In the randomised phase of the trial, children and adolescents were followed-up until the last patient reached 96 weeks of follow-up (appendix p 17).

First-line antiretroviral therapy included a third agent and two nucleoside or nucleotide reverse transcriptase inhibitors (NRTIs). Second-line antiretroviral therapy included a new third agent and two NRTIs, with at least one NRTI preserving activity as assessed on the basis of resistance tests or assumed from treatment history (when resistance tests were not routinely available). ODYSSEY included pharmacokinetic substudies evaluating simplified dolutegravir dosing.^20,21^ Once the results from these substudies were available, children assigned to dolutegravir received higher doses (50 mg film-coated tablets for children weighing ≥20 kg and 25 mg dispersible [five 5 mg tablets] for children weighing 14 kg to <20 kg; appendix p 6). Antiretroviral drugs in the standard-of-care group and NRTIs in the dolutegravir group were administered per national treatment guidelines.

Neuropsychiatric adverse events were captured through clinician-reported adverse events. At clinic visits, clinicians ascertained all adverse events since the last visit and reported adverse events of grade 3 or above, serious adverse events, adverse events leading to treatment modification, and notable events, which included suicidality events of any grade (suicidal ideation or behaviour). Identified events meeting reporting criteria were reported in case report forms. All reported events were reviewed by an independent endpoint review committee whose members were masked to group assignment.

Mood and sleep questionnaires were introduced after the first protocol amendment, approximately 10–12 months after the trial start, as neuropsychiatric safety concerns and sleep issues became apparent in adults. Paper-based questionnaires were administered at weeks 0, 4, 12, and 24, and every 24 weeks thereafter to participants or carers (or both) once the participant was aged 6 years or older. Carers accompanying the participants during the trial visits were also requested to complete the questionnaires. The questionnaires were designed in-house. We focused on a few key questions to avoid overburdening participants and their carers (appendix p 5). Mood questions aimed to identify symptoms of anxiety, low mood, self-harm, and suicidal thoughts experienced by the child in the past month: “low mood or feeling sad often”, “feeling worried often”, “feeling angry or aggressive often”, “hurting or harming himself/herself”, “thinking life was not worth living”, and “thoughts about ending life”. Questions about occurrence of dizziness and concentration difficulties were included in the questionnaires to evaluate common neurological symptoms in children. Questions for sleep assessment were adapted from the Pittsburgh questionnaire,^22^ focusing on key questions relevant to children. These questions included time taken to fall asleep, frequency of waking up during the night, frequency of nightmares or vivid dreams, and frequency of “trouble staying awake at school or during everyday activities in the past month”. Overall sleep quality in the past month was assessed using smiley face Likert scales with added verbal descriptions of very good, good, fair, not that good, or very bad.

### Outcomes

The primary outcome of the ODYSSEY trial, published previously, was the proportion of participants with virological or clinical treatment failure by 96 weeks (appendix p 20). In this Article, we report findings of two additional pre-specified outcomes: neuropsychiatric adverse events and responses to the mood and sleep questionnaires (appendix pp 20, 32). Neuropsychiatric adverse events were defined as adverse events of the nervous system (excluding adverse events of infectious causes) or psychiatric disorders diagnosed by treating clinicians or referred specialists, or both. Serious adverse events and adverse events of grade 3 or above were prespecified in the trial’s secondary outcomes. Suicidal ideation or behaviour of any grade were notable events in the trial. Any reported anxiety, low mood, self-harm, and suicidal thoughts on a mood questionnaire, as described, were included in the analysis. A sleep issue was defined as: taking 30 min or more to get to sleep; occasionally or frequently having nightmares or vivid dreams (or both), being awake at night, or having trouble staying awake during the day; and sleep quality being not that good or very bad.

### Statistical analysis

The statistical analysis plan can be found in the appendix (pp 13–35). Adverse events were compared between treatment groups by the proportion of participants with at least one event using the χ^2^ test and by time to first event using a Cox model, with adjustment for ODYSSEY A versus ODYSSEY B in the intention-to-treat population. Adverse event rates (per 100 person-years) were calculated as: the number of events/total person-years at risk×100 (with exact Poisson-based 95% CI). In an exploratory analysis, event rates were compared between participants in the dolutegravir group and participants receiving efavirenz or other third agents in the standard-of-care group using a Poisson model, adjusted for clustering by participant (ODYSSEY A *vs* ODYSSEY B was not adjusted for as the treatment-line was strongly associated with the standard-of-care regimen). Suppression at neuropsychiatric event was evaluated with a viral load of less than 400 copies per mL and using the result of the viral load closest to the date of diagnosis.

For mood and sleep analyses, the worst of the carer and participant report was taken at each study visit when both were available. Proportions of participants ever reporting a mood or sleep issue on the questionnaires were compared by treatment groups using the Mantel Haenszel χ^2^ test, stratified by ODYSSEY A versus ODYSSEY B. Ordered logistic mixed models were used to compare the outcomes in sleep questionnaires across treatment groups over time with a random effect for intercept and fixed effects for treatment group, study visit, and randomisation stratification factors (ODYSSEY A *vs* ODYSSEY B, abacavir and lamivudine NRTI backbone *vs* other NRTI backbones, routine resistance testing available *vs* unavailable). Analyses were performed using STATA software, version 16.1.

### Role of the funding source

ViiV Healthcare reviewed and commented on the manuscript. Employees of the Medical Research Council Clinical Trials Unit at University College London and the Penta Foundation had a role in the study design, data collection, data analysis, data interpretation, and writing of the manuscript.

## Results

Between Sept 20, 2016, and June 22, 2018, 707 participants were enrolled, of whom 345 (49%) were female and 362 (51%) were male. 623 (88%) of 707 participants were Black-African. 350 (49%) of 707 participants were randomly assigned to receive dolutegravir-based antiretroviral therapy and 357 (50%) to non-dolutegravir-based standard-of-care. At baseline, median age was 12·2 years (IQR 9·1–14·9; range 2·9–18·0) and 189 (27%) of 707 participants had a history of advanced HIV (WHO stage 3 or above).

By design, 311 (44%) of 707 participants starting first-line antiretroviral therapy were enrolled in ODYSSEY A and 396 (56%) starting second-line antiretroviral therapy were enrolled in ODYSSEY B. Most participants who were randomly assigned to the standard-of-care group initiated efavirenz-based antiretroviral therapy in ODYSSEY A (145 [92%] of 157) or protease inhibitor-based antiretroviral therapy in ODYSSEY B (195 [98%] of 200). Of 707 participants, 463 (65%) received abacavir plus lamivudine, 162 (23%) received tenofovir disoproxil fumarate plus lamivudine or emtricitabine, 77 (11%) received zidovudine plus lamivudine, and five (1%) received other backbones; backbones were balanced between treatment groups (table 1). Participants were followed-up until the last participant reached 96 weeks of follow-up (median 142 weeks [IQR 124–159]). A total of 50 (7%) of 707 participants were lost to follow-up.

Overall, 23 participants (15 in the dolutegravir group and eight in the standard-of-care group) had 31 neuropsychiatric adverse events (difference in proportion of participants with at least one event p=0·13). Median age at first event was 15·9 years (IQR 10·4–17·5) and median time from enrolment to first event was 72 weeks (47–124). 11 participants (six in the dolutegravir group and five in the standard-of-care group) had one or more neurological events (p=0·74). 14 participants (ten and four) had one or more psychiatric events (p=0·097; table 2). 19 (83%) of 23 participants with neuropsychiatric events had no reported history of pre-trial neuropsychiatric conditions or symptoms, although this was not systematically ascertained (appendix p 8). 20 (87%) of 23 participants were suppressed with a viral load of less than 400 copies per mL at the time of all their neuropsychiatric events (appendix pp 7–8).

Of 11 participants with neurological adverse events (nine male and two female), eight were in ODYSSEY A (four in the dolutegravir group and four in the standard-of-care group) and three were in ODYSSEY B (two and one). Median age at first event was 10·4 years (IQR 7·5–15·0) and median time from enrolment to first event was 68 weeks (19–126). Four participants in each treatment group had epileptic convulsions or seizures. Of the four participants in the standard-of-care group, one had status epilepticus due to ongoing uncontrolled epilepsy, 17 days after switching from efavirenz to dolutegravir for treatment failure. Other events included dizziness (reported twice in one participant in standard-of-care group), headache associated with hypertension (one in the dolutegravir group), and dystonia (one in the dolutegravir group). The participant with dystonia (thought to be caused by antipsychotics prescribed for psychosis, see below) had discontinued dolutegravir 95 weeks before the event. Efavirenz dose was reduced in the participant with dizziness because of the first episode. There were no other antiretroviral therapy modifications due to neurological events (appendix pp 7–8). All participants were receiving the abacavir plus lamivudine backbone at the time of the events.

Of 14 participants with psychiatric adverse events (nine male and five female), ten were in ODYSSEY A (eight in the dolutegravir group and two in the standard-of-care group) and four were in ODYSSEY B (two and two). Median age at first event was 17·2 years (IQR 15·4–18·5) and median time from enrolment to first event was 84 weeks (50–107). Eight participants in the dolutegravir group had suicidal ideation or behaviour (including one with depression), and four participants in the standard-of-care group had suicidal ideation or behaviour (including two with >1 suicidality-related event). Other events in the dolutegravir group included a diagnosis of depression without suicidal ideation or behaviour (n=1) and psychosis and insomnia (n=1). The patient with depression (without suicidal ideation or behaviour) was receiving efavirenz at the time of the event, having stopped dolutegravir 68 weeks earlier due to a hypersensitivity reaction and modified antiretroviral therapy again due to depression (changing to rilpivirine). The participant with psychosis and insomnia stopped dolutegravir following psychosis and had been taking nevirapine for 95 weeks at the time of insomnia (and dystonia). One participant in the standard-of-care group taking efavirenz modified their treatment following suicidal ideation (appendix p 8). At the time of the events, eight participants were receiving abacavir plus lamivudine backbone (six in the dolutegravir group and two in the standard-of-care group), five were receiving tenofovir plus lamivudine or emtricitabine (four and one), and one was receiving zidovudine plus lamivudine (one in the standard-of-care group).

In a post-hoc exploratory analysis, we compared the rate of neuropsychiatric events in participants receiving dolutegravir in the dolutegravir group with that in those receiving efavirenz in the standard-of-care group (incidence rate ratio 0·80, 95% CI 0·26–2·47; p=0·69) and with the rate in those receiving other third agents in the standard-of-care group (1·80, 0·56–5·72; p=0·32). Most neuropsychiatric events in the dolutegravir group were in participants weighing 40 kg or more, for whom the adult dolutegravir dose was used throughout the trial. Participants in the dolutegravir group who weighed less than 40 kg mostly started on the earlier licensed dolutegravir doses, which were subsequently increased (appendix p 6) on the basis of the results of nested pharmacokinetic substudies within the trial.^20,21^ There was no evidence of an increased rate of neuropsychiatric events with the approved increased doses of dolutegravir (table 3). Five (31%) of 16 neuropsychiatric events occurring during treatment with dolutegravir (including one event in the standard-of-care group) and four (50%) of eight occurring during treatment with efavirenz (including one event in the dolutegravir group) were considered at least possibly related to the antiretroviral therapy regimen by the endpoint review committee. None of the events on non-dolutegravir and non-efavirenz regimens were considered at least possibly related.

There was no difference between randomised groups in the proportions of participants with a report of “low mood or feeling sad often”, “feeling worried often”, and “feeling angry or aggressive often” at one or more follow-up visits. More participants or carers in the dolutegravir group than the standard-of-care group reported symptoms of self-harm (eight in the dolutegravir group and one in the standard-of-care group; p=0·025), life not worth living (17 and five; p=0·0091), or suicidal thoughts (13 and none; p=0·0006) at one or more follow-up visit (table 4). The results were consistent in ODYSSEY A and ODYSSEY B (appendix pp 9–10).

Among participants with reports of “life was not worth living” or suicidal thoughts, most (19 [83%] of 23 in the dolutegravir group and all five in the standard-of-care group) had only one report. Of 23 participants in the dolutegravir group with reports of either symptom, six (26%) had reports at the time or before a psychiatric event. None of the five participants in the standard-of-care group who reported these symptoms on the questionnaires had psychiatric events.

More participants in the standard-of-care group had reports of dizziness than those in the dolutegravir group (table 4), with consistent results in ODYSSEY A (31 [20%] of 153 in the dolutegravir group and 51 [34%] of 151 in the standard-of-care group; p=0·0078) and ODYSSEY B (33 [17%] of 195 and 48 [24%] of 197; p=0·069; appendix p 9). The numbers of participants with problems concentrating were similar in both groups (table 4).

We found no difference in the comparison of time to fall to sleep, reported nightmares or vivid dreams, frequency of waking up at night, trouble staying awake during the day, or sleep quality by treatment group, and no difference in proportions of participants with a reported sleep issue at one or more follow-up visits (table 5). Similar results were seen in children in ODYSSEY A and B (appendix pp 11–12).

## Discussion

This is the first study presenting comparative data on neuropsychiatric manifestations in children and adolescents treated with dolutegravir-based antiretroviral therapy versus non-dolutegravir containing standard-of-care regimens from a randomised controlled trial. Over a median follow-up of 142 weeks, few participants had a neuropsychiatric adverse event. Numerically, more participants had psychiatric events in the dolutegravir group, although the numbers were low. Additionally, more participants reported suicidal thoughts in mood questionnaires in the dolutegravir group than the standard-of-care group; most participants reported this outcome only once with no reports in subsequent visits.

The proportion of children experiencing neuro-psychiatric adverse events in the dolutegravir group in ODYSSEY was consistent with other studies of children receiving dolutegravir, in which proportions ranged from 1% to 7% of exposed patients.^14–18^ Our results are also in line with findings from meta-analyses of trials in adults.^6,9,10^ The meta-analysis by Hill and colleagues^10^ found that the risk of suicidality events in nine randomised controlled trials (5439 randomised participants who were either naive or exposed to antiretroviral therapy) was similar in participants receiving dolutegravir-based and non-dolutegravir-based regimens (relative risk [RR] 1·21, 95% CI 0·59–2·47). The difference remained non-significant when efavirenz, previously linked to increased suicide risk, was excluded from non-dolutegravir regimens (1·58, 0·56–4·45). Reeves and colleagues^9^ later reported the results of an updated meta-analysis of 14 randomised controlled trials with more than 10 000 adults who were either naive or previously exposed to antiretroviral therapy, and found no difference in depression, anxiety, and suicidality among participants randomised to dolutegravir-based, three-drug, antiretroviral therapy compared with non-dolutegravir-based treatment. However, compared with non-efavirenz-based antiretroviral therapy, dolutegravir-based therapy showed a potential association with depressive symptoms (1·26, 0·96–1·65).^9^ In a large network meta-analysis of 68 randomised controlled trials with more than 23 000 participants naive to antiretroviral therapy, there were no differences in neuropsychiatric adverse events between dolutegravir and most other anchor drugs.^6^ However, dolutegravir was associated with fewer neuropsychiatric adverse events than efavirenz 600 mg (odds ratio 0·36, 95% CI 0·16–0·82) or efavirenz 400 mg (0·32, 0·10–0·98), and fewer suicidality adverse events than ritonavir-boosted darunavir (0·12, 0·02–0·79).^6^ Similar to other studies, we showed that dolutegravir might be associated with a lower risk of neuropsychiatric events than efavirenz and a higher risk of neuropsychiatric events than non-efavirenz-based regimens, although we had few events, and the differences were not significant.

In ODYSSEY, we found no difference in sleep problems or overall quality of sleep by trial group. These results are consistent with the meta-analyses by Reeves and colleagues^9^ and Kanters and colleagues,^6^ which also showed no difference in insomnia in participants who received dolutegravir compared with other antiretroviral drugs. Although Hill and colleagues^10^ showed an increased risk of grade 1–4 insomnia with dolutegravir compared with other antiretroviral anchor drugs (RR 1·30, 95% CI 1·04–1·63), the relative risk was low. In ODYSSEY, fewer participants reported dizziness in questionnaires in the dolutegravir group than in the standard-of-care group. These results were consistent in children starting first-line or second-line treatment, with most receiving first line in the standard-of-care group taking efavirenz (92%) and nearly all on second line taking ritonavir-boosted protease inhibitors (98%). Meta-analyses of studies in adults have shown a lower risk of dizziness with dolutegravir than with efavirenz,^6^ or in control groups including efavirenz,^9^ but no differences between dolutegravir and non-efavirenz anchor agents.^6,9^

Real-world cohort studies have reported slightly higher rates of neuropsychiatric adverse events than in randomised controlled trials.^11^ These differences might be due to under-representation of individuals at high risk of neuropsychiatric adverse events in clinical trials, short duration of trial follow-up, limiting data collection to high-impact adverse events (eg, high severity grade, events leading to treatment discontinuation, or drug-related events), and reporting adverse events of lower grades only if they occur above a certain frequency threshold (eg, ≥5% of patients). Channelling bias could have occurred in early adult cohorts, when patients with pre-existing neuropsychiatric conditions, who were at higher risk of neuropsychiatric adverse events, were more likely to receive dolutegravir than efavirenz (known to be associated with neuropsychiatric toxicities).^23^ In later cohorts, clinician awareness of dolutegravir-associated neuropsychiatric adverse events might have contributed to identifying more events with dolutegravir and increased rates of discontinuations.^11,24^

Some dolutegravir cohort studies in adults found a higher incidence of neuropsychiatric adverse events in female participants than male participants, as well as among those receiving abacavir compared with those receiving antiretroviral therapy without abacavir.^11,25^ In ODYSSEY, few neuropsychiatric adverse events occurred but the proportions of participants with events were similar in female and male participants and among those who started abacavir and tenofovir.

There is emerging evidence that neuropsychiatric symptoms related to dolutegravir might resolve or improve with its discontinuation.^26^ In ODYSSEY, only one child discontinued dolutegravir due to a neuropsychiatric event (psychosis), with no improvement after discontinuation. Rare discontinuations might be attributed to inadequate alternative options from available anchor antivirals in low-income and middle-income countries; efavirenz is known to be associated with neuropsychiatric adverse events, and ritonavir-boosted protease inhibitors have drug interactions with many antipsychotic and antiepileptic drugs that patients with neuropsychiatric manifestations might receive.

Underlying mechanisms of dolutegravir-related neurotoxicity are not fully understood. Dolutegravir distributes well into CSF,^27^ which is postulated to link to neurotoxicity. Dose-related toxicity was suggested by a small Japanese study (n=162), which showed positive association between dolutegravir plasma exposure (C_trough_) and neuropsychiatric adverse events,^28^ although this finding was not confirmed in a large cohort study from Germany (n=861).^29^ VIKING-3, a randomised controlled trial evaluating double-dosing of dolutegravir in adults with integrase inhibitors-resistant HIV, also showed no increased neuropsychiatric adverse events with double-dose dolutegravir despite average dolutegravir trough concentrations being two times higher than that reported with the 50 mg once-daily regimen.^30^ In ODYSSEY, although we did not assess dolutegravir drug concentrations at the onset of adverse events, we had no indication that children receiving higher dolutegravir doses experienced higher rates of neuropsychiatric events than those on the initial reduced approved dosing.

The main strength of ODYSSEY is that it was a pragmatic global trial, and children with pre-existing neuropsychiatric conditions were eligible for enrolment. We had excellent retention with only 7% of participants lost to follow-up.^7^ The main limitation is that ODYSSEY was an open-label study and therefore could be prone to identification bias of neuropsychiatric adverse events as previously mentioned. Site clinicians were trained to ask for new events since the last visit, but we did not have a pre-specified symptom check for neuropsychiatric events, so the ascertainment of adverse events might have differed between clinicians. We also did not use validated suicidality questionnaires, like the Columbia-Suicide Severity Rating Scale protocol, and therefore might have underestimated the incidence of suicidal ideation and behaviour not identified by in-house questionnaires. However, our designed in-house short questionnaires included two questions related to suicidality ideation, allowing clinicians to explore this further during the study visits.

Overall, the number of neuropsychiatric adverse events and reported neuropsychiatric symptoms in the ODYSSEY trial were low. However, numerically more participants in the dolutegravir group than in the standard-of-care group had psychiatric events and reported suicidality ideation. These differences should be interpreted with caution in an open-label trial when previous knowledge of the associated adverse reactions with the received drugs could influence reported symptoms. Clinicians and policy makers should be aware of psychiatric manifestations in children and adolescents receiving dolutegravir and consider including suicidality screening in routine clinical reviews to identify patients at risk.

## Supplementary Material

Supplementary appendix

## Figures and Tables

**Table 1:** Baseline characteristics

	Dolutegravir group(n=350)	Standard of care group (n=357)
Country or region		
Uganda	170 (49%)	161 (45%)
Zimbabwe	79 (23%)	67 (19%)
South Africa	61 (17%)	83 (23%)
Thailand	28 (8%)	33 (9%)
Europe	12 (3%)	13 (4%)
Sex		
Female	174 (50%)	171 (48%)
Male	176 (50%)	186 (52%)
Age at randomisation, years	12·2 (9·2–15·1; 3·4–18·0)	12·1 (8·8–14·7; 2·9–18·0)
Weight, kg	30·4 (23·7–43·7; 14·0–85·0)	31·0 (23·3–42·7; 14·2–72·7)
Ethnic origin		
Black-African	310 (89%)	313 (88%)
Asian	28 (8%)	32 (9%)
White	5 (1%)	1 (<1%)
Other	7 (2%)	11 (3%)
CD4%*	20 (11–29)	23 (13–31)
CD4, cells per mm^3^*	444 (197–653)	487 (254–751)
Log_10_ HIV viral load, copies per mL*†	4·5 (3·9–5·1)	4·4 (3·7–4·9)
History of WHO staging		
Stage 1–2	253 (72%)	265 (74%)
Stage 3	69 (20%)	60 (17%)
Stage 4	28 (8%)	32 (9%)
Initial ART regimen: NRTI backbone		
Abacavir + lamivudine	232 (66%)	231 (65%)
TDF + lamivudine or emtricitabine	80 (23%)	82 (23%)
Zidovudine + lamivudine	37 (11%)	40 (11%)
Other‡	1 (0%)	4 (1%)
Initial ART regimen: third agent		
Dolutegravir	350 (100%)	··
Efavirenz	··	150 (42%)
Lopinavir + ritonavir	··	147 (41%)
Atazanavir + ritonavir	··	49 (14%)
Other§	··	11 (3%)

Data are n (%), median (IQR; range), or median (IQR). ART=antiretroviral therapy. NRTI=nucleoside or nucleotide reverse transcriptase inhibitor. TDF=tenofovir disoproxil fumarate.

* Mean measurement at screening and randomisation if both were available.

† One participant missing baseline viral load.

‡ Other initial NRTIs: three participants started abacavir plus tenofovir disoproxil fumarate (one in the dolutegravir group, two in the standard-of-care group), two started tenofovir alafenamide plus emtricitabine (two in the standard-of care group).

§ Other third agents in the standard-of-care group: six participants started ritonavir-boosted darunavir, one elvitegravir, two nevirapine, and two rilpivirine.

**Table 2:** Neuropsychiatric adverse events

	Dolutegravir group (n=350)	Standard-of-care group (n=357)	p value
Median follow-up, weeks	142 (123–159)	142 (123–158)	··
Neuropsychiatric adverse events	18 (n=15)	13 (n=8)	0·13*
Serious adverse events	7 (n=5)	6 (n=5)	··
ART-modifying adverse events†	2 (n=2)	2 (n=2)	··
Hazard ratio for time to first neuropsychiatric adverse event‡	1·87 (0·79–4·41)	1 (ref)	0·15
Neurological adverse events	6 (n=6)	6 (n=5)	0·74*
Epilepsy convulsions or seizures	4 (n=4)	4 (n=4)	··
Dizziness	0 (n=0)	2 (n=1)	··
Headache with hypertension	1 (n=1)	0 (n=0)	··
Dystonia	1 (n=1)	0 (n=0)	··
Hazard ratio for time to first neurological adverse event‡	1·18 (0·36–3·87)	1 (ref)	0·78
Psychiatric adverse events	12 (n=10)	7 (n=4)	0·097*
Suicidal ideation or behaviour	8 (n=8§)	7 (n=4¶)	··
Depression	2 (n=2§)	0 (n=0)	··
Insomnia	1 (n=1||)	0 (n=0)	··
Psychosis	1 (n=1||)	0 (n=0)	··
Hazard ratio for time to first psychiatric adverse event‡	2·48 (0·78–7·90)	1 (ref)	0·13

Data are median (IQR), number of events (number of participants), or hazard ratio (95% CI). ART=antiretroviral therapy.

* Comparing proportion of participants with at least one event.

† One additional participant in the dolutegravir group changed antiretroviral therapy due to an ongoing neuropsychiatric adverse event after the trial censoring date.

‡ Hazard ratio adjusted for ODYSSEY A and B. Tests for interactions between ODYSSEY A and B and trial group (dolutegravir *vs* standard-of-care) were non-significant: p=0·60 for all neuropsychiatric events, p=0·61 for neurological events, and p=0·28 for psychiatric events.

§ Two events, parasuicide and depression, occurred in the same patient.

¶ Three events, two suicidal ideations and one parasuicide, occurred in one patient; two suicidal ideation events occurred in another patient.

|| Two events, insomnia and psychosis, occurred in the same patient.

**Table 3:** Neuropsychiatric events by treatment group and dolutegravir dosing

	Neurological adverse events	Psychiatric adverse events	Neuropsychiatric adverse events	Rate of neuropsychiatric adverse events per 100 person-years (95% CI)
Dolutegravir group*	6	12	18	1·91 (1·13–3·02)
Receiving dolutegravir	5	10	15	1·62 (0·91–2·68)
≥40 kg, approved 50 mg tablet	1	8	9	1·99 (0·91–3·77)
<40 kg, previously approved (reduced) doses†	2	1	3	1·70 (0·35–4·96)
<40 kg, approved (increased) doses‡	2	1	3	1·03 (0·21–3·00)
Receiving non-dolutegravir third agent§	1	2	3	20·17 (4·16–58·95)
Standard-of-care group¶	6	7	13	1·39 (0·74–2·38)
Receiving efavirenz	4	3	7	2·01 (0·81–4·14)
Receiving dolutegravir	1	0	1	13·13 (0·33–73·20)
Receiving alternative third agent||	1	4	5	0·88 (0·28–2·05)

* No events occurred in participants in the dolutegravir group who were receiving the non-protocol dolutegravir dose (follow-up 0·74 person years) or were not receiving antiretroviral therapy or a third agent (follow-up 4·39 person years).

† Reduced dolutegravir doses: the ODYSSEY trial opened with children receiving doses evaluated by the IMPAACT dose-finding study or approved by the US Food and Drug Administration or European Medicines Agency (20 mg film-coated tablets in participants weighing 15–<20 kg, 25 mg film-coated tablets in those weighing 20–<30 kg, and 35 mg film-coated tablets in those weighing 30–<40 kg); participants weighing 14–<20 kg in the pharmacokinetic substudy received 25 mg.

‡ Following results from the ODYSSEY nested weight-band pharmacokinetic substudy,^20,21^ children were moved to increased dolutegravir doses: 25 mg dispersible tablets in participants weighing 14–<25 kg and 50 mg film-coated tablets in those weighing 20–<40 kg. The US Food and Drug Administration and European Medicines Agency dosing licenses were subsequently updated; dispersible tablets have 1·6–1·8 times higher bioavailability than film-coated tablets.

§ In the dolutegravir group, two neuropsychiatric adverse events (one neurological event and one psychiatric event) occurred in the same participant while taking nevirapine and one psychiatric event occurred in a participant taking efavirenz.

¶ No events occurred in participants in the standard-of-care group who were not receiving antiretroviral therapy or third agent (follow-up 6·52 person years).

|| In the standard-of-care group, three neuropsychiatric adverse events occurred in participants receiving ritonavir-boosted lopinavir (one neurological event and two psychiatric events) and two psychiatric events occurred in participants receiving ritonavir-boosted atazanavir.

**Table 4:** Reported symptoms on mood questionnaire

	Dolutegravir group (n=350)	Standard- of-care group (n=357)	p value*
Follow-up questionnaires (participants completing ≥1 questionnaire)†	2073 (348)	2011 (348)	··
Dizziness			
Total reports during follow-up (all follow-up questionnaires)	92 (4%)	141 (7%)	··
Participants reporting issue at ≥1 follow-up visit	64 (18%)	99 (28%)	0·0015
Problems concentrating			
Total reports during follow-up (all follow-up questionnaires)	38 (2%)	51 (3%)	··
Participants reporting issue at ≥1 follow-up visit	33 (9%)	41 (12%)	0·32
Low mood			
Total reports during follow-up (all follow-up questionnaires)	137 (7%)	142 (7%)	··
Participants reporting issue at ≥1 follow-up visit	83 (24%)	95 (27%)	0·28
Worried often			
Total reports during follow-up (all follow-up questionnaires)	53 (3%)	57 (3%)	··
Participants reporting issue at ≥1 follow-up visit	44 (13%)	46 (13%)	0·82
Angry or aggressive often			
Total reports during follow-up (all follow-up questionnaires)	140 (7%)	149 (7%)	··
Participants reporting issue at ≥1 follow-up visit	89 (26%)	87 (25%)	0·86
Self-harm			
Total reports during follow-up (all follow-up questionnaires)	8 (<1%)	1 (<1%)	··
Participants reporting issue at ≥1 follow-up visit	8 (2%)	1 (<1%)	0·025
Life not worth living			
Total reports during follow-up (all follow-up questionnaires)	20 (1%)	5 (<1%)	··
Participants reporting issue at ≥1 follow-up visit	17 (5%)	5 (1%)	0·0091
Suicidal thoughts			
Total reports during follow-up (all follow-up questionnaires)	13 (1%)	0 (0%)	··
Participants reporting issue at ≥1 follow-up visit	13 (4%)	0 (0%)	0·0006

Data are n (%) unless specified otherwise. The questionnaires explored symptoms in the past month. The worst of the carer-reported and participant-reported mood symptom was taken at each study visit.

* Mantel-Haenszel χ^2^ test.

† Questionnaires were completed for participants aged 6 years or older at visit.

**Table 5:** Reported sleep problems and overall quality of sleep

	Dolutegravir group (n=350)	Standard-of-care group (n=357)	p values
Follow-up questionnaires (participants completing ≥1 questionnaire)*	2162 (349)	2096 (350)	··
Time taken to fall asleep	··	··	0·51†
<15 mins	1531 (71%)	1490 (71%)	··
≥15–30 mins	496 (23%)	451 (22%)	··
30–60 mins	90 (4%)	85 (4%)	··
>1 h	19 (1%)	31 (1%)	··
Unknown	20 (1%)	27 (1%)	··
Participants reporting issue at ≥1 follow-up visit‡	76 (22%)	75 (21%)	0·91§
Awake during the night	··	··	0·75†
Never	1738 (80%)	1724 (82%)	··
Infrequently	218 (10%)	171 (8%)	··
Occasionally	106 (5%)	96 (5%)	··
Frequently	88 (4%)	86 (4%)	··
Unknown	10 (<1%)	19 (1%)	··
Participants reporting issue at ≥1 follow-up visit‡	126 (36%)	131 (37%)	0·71§
Trouble staying awake during the day	··	··	0·49†
Never	1841 (85%)	1753 (84%)	··
Infrequently	142 (7%)	147 (7%)	··
Occasionally	90 (4%)	97 (5%)	··
Frequently	79 (4%)	81 (4%)	··
Don’t know	7 (<1%)	16 (1%)	··
Participants reporting issue at ≥1 follow-up visit‡	105 (30%)	111 (32%)	0·64§
Nightmares or vivid dreams, or both	··	··	0·11†
Never	1360 (63%)	1375 (66%)	··
Infrequently	311 (14%)	252 (12%)	··
Occasionally	271 (13%)	261 (12%)	··
Frequently	155 (7%)	143 (7%)	··
Unknown	63 (3%)	65 (3%)	··
Participants reporting issue at ≥1 follow-up visit‡	194 (56%)	178 (51%)	0·21§
Sleep quality	··	··	0·89†
Very good	672 (31%)	656 (31%)	··
Good	1349 (62%)	1326 (63%)	··
Fair	104 (5%)	86 (4%)	··
Not that good	26 (1%)	22 (1%)	··
Very bad	9 (<1%)	6 (<1%)	··
Participants reporting issue at >1 follow-up visit*	30 (9%)	24 (7%)	0·39§

Data are total number of reports (% of all follow-up questionnaires), unless specified otherwise. The questionnaires explored sleep in the past month. The worst of carer-reported and participant-reported sleep description was taken at each study visit.

* Questionnaires completed for participants aged 6 years or older at visit.

† Ordered logistic mixed models are used to compare outcomes across treatment groups over time with a random effect for intercept and fixed effects for treatment group, study visits, and adjustment stratification factors.

‡ Issue defined as 30 mins or more to get to sleep; occasionally or frequently being awake in the night, trouble staying awake during the days, or having nightmares or vivid dreams, or both; and sleep quality being not that good or very bad.

§ Mantel-Haenszel χ^2^ test.

## Research in context

### Evidence before the study

ODYSSEY, an open-label, randomised controlled trial, showed that dolutegravir-based antiretroviral therapy is more efficacious than other standard-of-care drugs and is safe for treating children with HIV starting first-line or second-line therapy. Dolutegravir has been widely rolled out for treating paediatric HIV. We searched PubMed from database inception to May 29, 2022, for studies on dolutegravir and neuropsychiatric manifestation, published in all languages, using the terms “central nervous system” OR “neurological” OR “neurotoxicity” OR “psychiatric” OR “neuropsychiatric” OR “sleep” OR “insomnia” AND “dolutegravir”. The search yielded 147 publications, including three meta-analyses of adult trials and 70 original research papers. Cohort studies in adults showed that dolutegravir was associated with neuropsychiatric manifestations and sleep problems leading to treatment discontinuation in around 4% of exposed individuals. Contrary to the cohort studies, three meta-analyses of randomised controlled trials in adults showed no increased risk of neuropsychiatric adverse events with dolutegravir compared with other anchor drugs; however, one of these meta-analyses showed a borderline increased risk of depressive symptoms with dolutegravir when efavirenz was excluded from comparators. Data on neuropsychiatric manifestations in children treated with dolutegravir-based antiretroviral therapy are scarce. A paediatric dolutegravir dose-finding study (IMPAACT P1093) reported two cases of psychiatric issues and some neurological events, mainly headache and dizziness. Four paediatric European cohort studies reported that 1–7% of participants, mainly adolescents, developed neuropsychiatric adverse events with dolutegravir-based antiretroviral therapy with rare dolutegravir discontinuations.

### Added value of this study

To our knowledge, this is the first study presenting comparative data on neuropsychiatric manifestations in children and adolescents treated with dolutegravir-based treatment versus non-dolutegravir standard-of-care regimens. Consistent with findings from other paediatric studies, a small proportion of children and adolescents (3%) developed neuropsychiatric adverse events, resulting in treatment changes in less than 1% of participants. There was no difference in the risk of neurological adverse events between the dolutegravir and standard-of-care treatment groups. However, in the dolutegravir group, numerically more children had psychiatric events than in the standard-of-care group (ten *vs* four), and more children reported that life was not worth living or suicidal thoughts. We found no differences between treatment groups in sleep problems or sleep quality.

### Implications of all the available evidence

Together with adult and other paediatric data, this study shows that neuropsychiatric adverse events with dolutegravir-based regimens are infrequent. Psychiatric manifestations in individuals receiving dolutegravir might be slightly higher than with other anchor drugs. Among paediatric patients, events predominantly occur in adolescents. Clinicians should be aware of the risk of psychiatric manifestations in children and adolescents taking dolutegravir and consider suicidality screening in routine consultations to identify patients for whom alternative antiretroviral therapy options could be considered.
