# Supplementary material for: Neuropsychiatric manifestations and sleep disturbances with dolutegravir-based antiretroviral therapy versus standard of care in children and adolescents: a secondary analysis of the ODYSSEY trial
Source: Lancet Child Adolesc Health. Author manuscript; Available in PMC 2024 Aug 8. (PMC7616346; doi:10.1016/S2352-4642(23)00164-5)
Supplement: Supplementary appendix [file EMS197915-supplement-Supplementary_appendix.pdf]

THE LANCET
Child & Adolescent Health

Supplementary appendix

This appendix formed part of the original submission and has been peer reviewed. We post it as supplied by the authors.

Supplement to: Turkova A, White E, Kekitiinwa AR, et al. Neuropsychiatric manifestations and sleep disturbances with dolutegravir-based antiretroviral therapy versus standard of care in children and adolescents: a secondary analysis of the ODYSSEY trial. *Lancet Child Adolesc Health* 2023; published online Aug 7. https://doi.org/10.1016/S2352-4642(23)00164-5.

Supplementary Appendix

Table of Contents

ODYSSEY Trial Team 2

Figure S1. Mood and sleep questionnaire 5

Table S1. Dolutegravir dosing in ODYSSEY across different protocol versions 6

Table S2. Details of neuropsychiatric events 7

Table S3. Summary of participants mood-related symptoms by ODYSSEY A and ODYSSEY B 9

Table S4: Reported sleep problems and overall quality of sleep by ODYSSEY A and ODYSSEY B........ 11

ODYSSEY Statistical Analysis Plan........ 13

ODYSSEY Trial Team

Penta Foundation: Carlo Giaquinto, Tiziana Grossele, Daniel Gomez-Pena, Davide Bilardi, Giulio Vecchia

Clinical Trial Units

MRC CTU at UCL: Shabinah S. Ali, Abdel Babiker, Shazia Begum, Chiara Borg, Anne-Marie Borges Da Silva, Joanna Calvert, Man Chan, Nimisha Dudakia, Deborah Ford, Joshua Gasa, Diana M. Gibb, Lily Houlden, Khadija Ibrahim, Nasir Jamil, Sarah Lensen, Emma Little, Fatima Mohamed, Samuel Montero, Cecilia L. Moore, Rachel Oguntimehin, Anna Parker, Reena Patel, Tasmin Phillips, Tatiana Sarfati, Karen Scott, Clare Shakeshaft, Moira Spyer, Margaret Thomason, Anna Turkova, Rebecca Turner, Nadine Van Looy, Ellen White, Ian White, Kaja Widuch, Helen Wilkes, Ben Wynne

INSERM SC-10-US19—ANRS: Alexandra Compagnucci, Yacine Saidi, Yoann Riault, Alexandra Coelho, Laura Picault, Christelle Kouakam

PHPT: Tim R. Cressey, Suwalai Chalermpantmetagul, Dujrudee Chinwong, Gonzague Jourdain, Rukchanok Peongjakta, Praornsuda Sukrakanchana, Wasna Sirirungsi

Trial sites

Joint Clinical Research Centre, Uganda: Cissy M. Kityo, Victor Musiime, Elizabeth Kaudha, Annet Nanduudu, Emmanuel Mujyambere, Paul Ocitti Labeja, Charity Nankunda, Juliet Ategeka, Peter Erim, Collin Makanga, Esther Nambi, Abbas Lugemwa, Lorna Atwine, Edridah Keminyeto, Deogratiuos Tukwasibwe, Shafic Makumbi, Emily Ninsiima, Mercy Tukamushaba, Rogers Ankunda, Ian Natuhurira, Miriam Kasozi, Baker Rubinga, Diana Antonia Rutebarika, Rashidah Nazzinda, Shamim Nakabuye, Julius Tumusiime, Alice Mulindwa, Ritah Mbabazi, Milly Ndigendawani, Edward Bagirigomwa, Eddie Rubanga, David Eram, Maria Nannungi, Chrispus Katemba, Disan Mulima, Josephine Namusanje, Mariam Nabalamba, Priscilla Kyobutungi, Phyllis Mwesigwa Rubondo, Robinah Kibenge, Claire Nasaazi, Basiimwa Roy Clark, Enock Babu, Alex Musiime, Faith Mbasani, Martin Ojok, Odoch Denis, David Baliruno, Katabalwa Juliet, Benson Ouma, Barbara Ainebyoona, Mariam Naabalamba, Diana A. Rutebarika, Alex V. Musiime, Josephine Kobusungye, Ezra Lutalo

Baylor College of Medicine Children's Foundation, Uganda: Adeodata R. Kekitiinwa, Pauline Amuge, Dickson Bbuye, Justine Nalubwama, Winnie Akobye, Muzamil Nsibuka Kisekka, Anthony Kirabira, Gloria Ninsiima, Sylvia Namanda, Gerald M. Agaba, Immaculate Nagawa, Annet Nalugo, Florence Namuli, Rose J. Kadhuba, Rachael K. Namuddu, Lameck Kiyimba, Angella Baita, Eunice Atim, Olivia Kobusingye, Clementine Namajja, Africanus Byaruhanga, Rogers Besigye, Herbert Murungi, Geoffrey Onen, Lawrence Lekku, Judith Tikabibamu, Henry Balwa

MUJHU Research Collaboration, Uganda: Philippa Musoke, Linda Barlow-Mosha, Grace Ahimbisibwe, Rosemary Namwanje, Hajira Kataike, Mark Ssenyonga, Brenda Kakayi, Rebecca Sakwa, Sarah Nakabuye, Barbara Musoke Nakirya, Gladys Kasangaki, Raymonds Kyambadde, David Balamusani, Winnie Nansamba, Stella Nalusiba, Emmanuel Mayanja, Richard Isabirye, Erinah Kyomukama, Rebecca Wampamba, Mildred Kabasonga, Zaam Zinda Nakawungu, Sarah Babirye, Olivia Kaboggoza, Juliet Nanyonjo, Joanita Nankya Baddokwaya, Alice Elwana, Winfred Kaahwa, Bosco Kafufu, Emmanuel Hakiza, Maria Musisi, Paula Namayanja, Maria Gorreti Nakalema, Robert Serunjogi, Monica Etima, Phionah Kibalama, Joel Maena, Agnes Mary Mugagga, Annet Miwanda, Monica Nolan.

UZCRC, Zimbabwe: James Hakim, Hilda Mujuru, Kusum Nathoo, Mutsa Bwakura-Dangarembizi, Ennie Chidziva, Shepherd Mudzingwa, Secrecy Gondo, Godfrey Musoro, Vivian Mumbiro, Gloria Tinago, Shirley Mutsai, Joy Chimanzi, Columbus Moyo, Ruth Nhema, Misheck Nkalo Phiri, Stuart Chitongo, Joshua Choga, Joyline Bhiri, Wilber Ishemunyoro, Makhosonke Ndlovu, Moses Chitsamatanga, Pia Ngwaru, Tsitsi Gwenzi, Wendy

Mapfumo, Dorothy Murungu, Trust Mukanganiki, Prosper Dube, Tapiwa Gwaze, Farai Matimba, Tawona Mudzviti, Zivai Mupambireyi, Sibusisiwe Weza, Cleopatra Langa, Sandra Musarurwa, Shamiso Gwande

FAM-CRU, South Africa: Mark F. Cotton, Anita Janse van Rensburg, Marlize Smuts, Catherine Andrea, Sumaya Dadan, Sonja Pieterse, Vinesh Jeaven, Candice Makola, George Fourie, Kurt Smith, Els Dobbels, Peter Zuidewind, Hesti Van Huyssteen, Mornay Isaacs, Georgina Nentsa, Thabisa Ncgaba, Candice MacDonald, Maria Bester, Wilma Orange, Ronelle Arendze, Mark Mulder, Lucille Malgraaf, Ashley Harley, Yasmeen Akhalwaya

PHRU, South Africa: Avy Violari, Nastassja Ramsagar, Afaaf Liberty, Ruth Mathiba, Mandisa Nyati, Haseena Cassim, Lindiwe Maseko, Nkata Kekane, Busi Khumalo, Mirriam Khunene, Noshalaza Sbisi, Jackie Brown, Tryphina Madonsela, Nokuthula Mbadaliga, Zaakirah Essack, Reshma Lakha, Aasia Vadee, Derusha Frank, Nazim Akoojee, Maletsatsi Monametsi, Gladness Machache, Yolandie Fourie, Anusha Nanan-kanjee, Juan Erasmus, Angelous Mamiane, Tseleng Daniel, Fatima Mayat, Nomfundo Maduna, Patsy Baliram, Sibongile Sithebe, Emily Lebotsa, Siphiwe Mkhize

Klerksdorp Tshepong Hospital Complex, South Africa: Ebrahim Variava, Modiehi Rakgokong, Dihedile Scheppers, Tumelo Moloantoa, Abdul Hamid Kaka, Tshepiso Masienyane, Akshmi Ori, Kgosimang Mmolawa, Pattamukkil Abraham.

Durban International Clinical Research Site, South Africa: Moherndran Archary, Rosie Mngqibisa, Rejoice Mosia, Sajeeda Mawlana, Rashina Nundlal, Penelope Madlala, Allemah Naidoo, Sphiwee Cebekhulu, Petronelle Casey, Subashinie Sidhoo, Minenhle Chikowore, Lungile Nyantsa, Sheleika Singh

AHRI, South Africa: Nigel Klein, Osee Behuhuma, Olivier Koole, Kristien Bird, Nomzamo Buthelezi, Mumsy Mthethwa, Gugu Gasa, Siva Danaviah and Theresa Smit

PHPT CTU: Tim R. Cressey, Suwalai Chalermpantmetagul, Gonzague Jourdain, Nicole Ngo Giang Huong, Dujrudee Chinwong, Chalermpong Saenjum, Rukchanok Peongjakta, Pra-ornsuda Sukrakanchana, Woottichai Khamduang, Laddawan Laomanit, Ampika Kaewbundit, Jiraporn Khamkon, Kanchana Than-in-at, Sanuphong Chailert, Worathip Sripaoraya, Nitinart.krueduangkam, Namthip Kruenual, Warunee Khamjakkaew, Soraya Klinprung, **Prapokklao Hospital:** Chaiwat Ngampiyaskul, Pisut Greetanukroh, Praechadaporn Khannak, Pathanee Tearsansern, Wanna Chamjamrat, **Phayao Hospital, Thailand:** Nuttawut Chanto, Thitiwat Thapwai, Khanungnit Thungkham, Patcharee Puangmalai, Chutima Ruklao, **Chiangrai Prachanukroh Hospital, Thailand:** Pradthana Ounchanum, Suwimon Khusuwan, Sukanda Denjanta, Yupawan Thaweesombat, Jutarat Thewsoongnoen, Kanyanee Kaewmamueng, Phakamas Kamboua, Supawadee Pongprapass (Sangjan), Warunee Srisuk, Areerat Kongponoi, Juthamas Limplertjareanwanich, **Nakornping Hospital, Thailand:** Suparat Kanjanavanit, Prattana Leenasirimakul, Chayakorn Saewtrakool, Pacharaporn Yingyong, Duangrat Chutima (Suwan), Raungwit Junkaew, Orapin Khatngam, Thannapat Chankun, **Khon Kaen Hospital, Thailand:** Ussanee Srirompotong, Patamawadee Sudsaard, Sookpanee Wimonklang, Turian Petpranee, **Mahasarakam Hospital, Thailand:** Sathaporn Na-Rajsima, Pattira Runarassamee, Nuananong Kunjaroenrut, Arttasid Udomvised, Tassawan Khayanchoomnoom, Watchara Meethaisong, Ketmookda Trairat

HIVNAT, Thailand: Thanyawee Puthanakit, Suvaporn Anugulruengkitt, Wipaporn Natalie Songtaweesin, Torsak Bunupuradah, Naruporn Kasipong, Sararut Chanthaburanun, Apicha Mahanontharit, Kesdao Nanthapisal, Thidarat Jupimai, Thornthun Noppakaorattanamanee, Chutima Saisaengjan

European Site Investigators: Goethe University Frankfurt**, Germany:** Stephan Schultze-Strasser, Christoph Königs , **UKE Eppendorf, Germany:** Robin Kobbe, Ulf Schulze-Sturm, Felicia Mantkowski, Cornelius Rau, **Heartlands Hospital, UK:** Steve Welch, Jacqui Daglish, Laura Thrasyvoulou, Kate Gandhi, Yvonne Vaughan-Gordon, **Great Ormand Street Hospital, UK:** Delane Shingadia, Sophie Foxall, Judith Acero, Malgorzata Pasko-Szcech, Jacquie Flynn, **St Mary's Hospital, UK:** Gareth Tudor-Williams, Amina Farhana Mehar, Caroline Foster, Sobia Mustafa, **Leicester Royal Infirmary, UK:** Srini Bandi, Jin Li, Jackie Philps, **Leeds General Infirmary, UK:** Sean O'Riordan, Dominique Barker, Richard Vowden, Maria Dowie **Kings College Hospital, UK:** Colin Ball Eniola

Nsirim, Kathleen McClaughlin, **Hospital 12 de Octubre, Spain:** India Garcia, Pablo Rojo Conejo, Cristina Epalza, Luis Prieto Tato, Maite Fernandez **Hospital La Paz, Spain:** Luis Escosa Garcia, Maria José Mellado Peña, Talia Sainz Costa, **Hospital San Joan de Déu, Spain:** Claudia Fortuny Guasch, Antoni Noguera Julian, Carolina Estepa, Elena Bruno, Patricia Mendez Garcia, Alba Murciano Cabeza, Biobanco Gregorio Maranon, Maria Angeles Muñoz Fernandez, Jose Luis Jimenez, Coral Gomez Rico, **Centro Materno-infantile do Norte, Portugal:** Laura Marques, Carla Teixeira, Alexandre Fernandes, Rosita Nunes, Helena Nascimento, Andreia Padrao, Joana Tuna, Helena Ramos, Ana Constança Mendes, Helena Pinheiro, Ana Cristina Matos

Local Site Monitors: Flavia Kyomuhendo, Sarah Nakalanzi, Cynthia Mukisa Williams, Leora Sewnarain, Ntombenhle Ngcobo, Deborah Pako, Nompumelelo Yende, Jacky Crisp, Marlize Smuts, Benedictor Dube, Precious Chandiwana, Winnie Gozhora, Thidarat Jumpimai

Substudies

PK substudies: David Burger, Pauline Bollen, Angela Colbers, Hylke Waalewijn, Tom Jacobs

Virology-immunology substudy: Nigel Klein, Eleni Nastouli, Anita De Rossi, Maria Angeles Munoz Fernandez, Carlota Miranda, Moira Spyer,

Social Science substudy and Youth Trial Board project: Janet Seeley, Sarah Bernays, Stella Namukwaya, Zivai Mupambireyi, Magda Conway, Lungile Jafta, Mercy Shibemba

Trial Committees

Independent Trial Steering Committee Members: Ian Weller, Elaine Abrams, Tsitsi Apollo, Polly Clayden, Valériane Leroy

Independent Data Monitoring Committee Members: Anton Pozniak, Jane Crawley, Rodolphe Thiébaut, Helen McIlleron

Endpoint Review Committee Members: Alasdair Bamford, Hermione Lyall, Andrew Prendergast, Felicity Fitzgerald, Anna Goodman

Figure S1. Mood and sleep questionnaire

SLEEP AND MOOD WITHIN THE LAST MONTH

For Week 0 Complete Sleep and Mood section (Q6-14) only

6. In the last month have you experienced any of the following? *Tick all that apply*

Dizziness or room spinning ☐	Low mood or feeling sad often ☐	Other 1: ☐(Give details).....................................
Problems concentrating ☐	Hurting/harming myself *(e.g. taking an overdose, cutting)* ☐	Other 2: ☐(Give details).....................................
Feeling worried often ☐	Thinking life is not worth living ☐	Other 3: ☐(Give details).....................................
Feeling angry or aggressive often ☐	Thinking about ending life (suicidal thoughts) ☐	None of the above ☐<br>Not applicable as less than 6 years of age ☐

7. In the last month, what time did you usually go to bed at night? *(24h)* h h m m

8. In the last month, how long did you usually take to fall asleep each night? *Tick one answer only*

Less than 15 minutes ☐ 15 minutes to half an hour ☐ Half an hour to an hour ☐ More than an hour ☐ I don't know ☐

9. In the last month, approximately how many hours of sleep did you get each night? ☐
Write the number of hours

		Never	Infrequently *(e.g. once or twice a month)*	Occasionally *(e.g. once or twice a week)*	Frequently *(e.g. 3 or more times a week)*	Don't know
10.	In the last month, how often did you wake during the night? *Tick one only*	☐	☐	☐	☐	☐
11.	In the last month, how often did you experience nightmares? *Tick one only*	☐	☐	☐	☐	☐
12.	In the last month, how often did you experience vivid dreams (not nightmares)? *Tick one only*	☐	☐	☐	☐	☐
13.	In the last month, how often have you had trouble staying awake at school or during everyday activities? *Tick one only*	☐	☐	☐	☐	☐

14. In the last month how would you rate your sleep quality overall? *Tick one answer only*

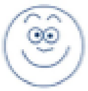
Very Good ☐

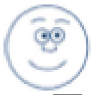
Good ☐

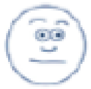
Fair ☐

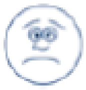
Not that good ☐

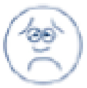
Very bad ☐

FOR ODYSSEY B ONLY

15. How do you find taking your antiretroviral medicine now compared to before starting ODYSSEY (when you were taking first-line treatment)? *Tick one answer only*

A lot easier ☐ A little easier ☐ No difference ☐ A little more difficult ☐ A lot more difficult ☐

THANK YOU FOR TAKING THE TIME TO FILL OUT THIS FORM

Table S1. Dolutegravir dosing in ODYSSEY across different protocol versions

	ODYSSEY v2.0*	ODYSSEY v3.0		ODYSSEY v4.0		ODYSSEY v5.0 onwards	
	Main trial participants	Main trial participants	WB PK substudy participants	Main trial participants	WB PK substudy participants	Main trial participants	WB PK substudy participants
3-<6kg	-	-		-	5mg or 10mg DT[ψ]	-	5mg or 10mg DT[ψ]
6-<10kg	-	-		-	15mg DT	-	15mg DT
10-<14kg	-	-		-	20mg DT	-	20mg DT
14-<15kg	-	-	25mg FCT	25mg FCT→25mg DT[§]	25mg DT	25mg DT	N/A
15-<20kg	20mg FCT*	20mg FCT					
20-<25kg	25mg FCT	25mg FCT	25mg FCT	25mg FCT→30mg DT or 50mg FCT[¥]	30mg DT or 50mg FCT†	50mg FCT[π]	N/A
25-<30kg	25mg FCT	25mg FCT→50mg FCT**	50mg FCT	25mg FCT→50mg FCT**	NA	50mg FCT	NA
30-<35kg	35mg FCT	35mg FCT→50mg FCT**	50mg FCT	35mg FCT→50mg FCT**	NA	50mg FCT	NA
35-<40kg	35mg FCT	35mg FCT→50mg FCT**	50mg FCT	35mg FCT→50mg FCT**	NA	50mg FCT	NA
≥40kg	50mg FCT	50mg FCT	NA	50mg FCT	NA	50mg FCT	NA

DT=dispersible tablets, FCT=film-coated tables

* In May 2017 the EMA licensed the use of 20mg DTG in children 15 - <20kg and ≥6years, following this, children were able to be recruited in this weight and age-band.

** From 1st of April 2018, after ethics notification, sites following protocol version 3.0 and above were recommended to increase the DTG dose of children 25 - <40kg to 50mg FCT QD at their next scheduled study visit based on the results of the WB-PK2. WB-PK2 participants remained on DTG 50mg with ongoing follow-up. Non-PK participants recruited after implementation were initiated on the 50mg film-coated DTG dose.

ψ Infants <6 months of age received DTG 5mg QD while infants ≥6 months of age received DTG 10mg QD, both as dispersible tablets.

† Both doses are examined in WB-PK1 part II substudy in this weight-band.

§ Children 15 - <20kg previously receiving DTG 20mg QD were changed to DTG film-coated 25mg tablets upon the approval of protocol v4.0. Subsequently all children 14-<20kg changed to DTG 25mg QD dispersible tablets following the review of WB-PK1 part I results and approval by the relevant ethical and regulatory authorities.

¥ Children 20 - <25kg previously receiving DTG 25mg QD as one 25mg film-coated tablet changed to either DTG 30mg QD dispersible tablets or DTG 50mg QD film-coated tablet (depending on site) following the review of WB-PK1 part I results and approval by the relevant ethical and regulatory authorities.

Π Following the review of PK and safety data children 20-<25kg receiving DTG 30mg dispersible tablets should be switched to DTG 50mg film-coated tablets. Those who prefer to remain on DTG 30mg DT will be able to do so until they move weight band.

Table S2. Details of neuropsychiatric events

Participant ID	Treatment arm	Sex	ODYSSEY A/B	Age at event	Week in the trial	ART regimen at the event onset	DTG weight-band dose and formulation at the event[§]	Viral load at the time of event, c/mL	Event name	DAIDS grade	SAE	ART-modi-fying	At least possibly related to ART[θ]
	**Neurological events**												
1	DTG	M	A	10	47	ABC 3TC DTG	25-<30kg, FCT 25mg	<40	Headache[¥]	4	yes	no	no
**2**	**DTG**	**M**	**A**	**14**	**130**	**ABC 3TC NVP[¤]<br>(on NVP from 35w)**		**15,000**	**Dystonia[ψ]**	**3**	**no**	**no**	**no**
3	DTG	M	B	7	68	ABC 3TC DTG	14-<20kg, DT 25mg	<20	Epilepsy, fits, convulsions[ψ]	4	yes[§]	no	no
4	DTG	M	A	4	19	ABC 3TC DTG	14-<20kg, FCT 25mg	<50	Epilepsy, fits, convulsions	3	no	no	no
5	DTG	M	B	10	95	ABC 3TC DTG	30-<40kg, FCT 50mg	<40	Epilepsy, fits, convulsions	3	no	no	no
6	DTG	M	A	14	2	ABC 3TC DTG	≥40kg, FCT 50mg BD[Ω]	64	Epilepsy, fits, convulsions	3	yes	no	no
**7**	**SOC**	**F**	**A**	**15**	**12**	**ABC 3TC EFV**		<20	**Dizziness**	**2**	**no**	**yes***	**yes**
**7**	**SOC**	**F**	**A**	**17**	**114**	**ABC 3TC EFV**		<20	**Dizziness**	**3**	**yes**	**no**	**yes**
8	SOC	M	A	10	132	ABC 3TC EFV		<40	Epilepsy, fits, convulsions	4	yes	no	no
9	SOC	M	B	16	124	ABC 3TC LPVr		50	Epilepsy, fits, convulsions	3	no	no	no
10	SOC	F	A	8	126	ABC 3TC DTG<br>(on DTG from 124w)		<40	Epilepsy, fits, convulsions[ψ]	4	yes	no	no
**11**	**SOC**	**M**	**A**	**7**	**50**	**ABC 3TC EFV**		**<20**	**Epilepsy, fits, convulsions**	**3**	**yes**	**no**	no
	**Psychiatric events**												
12	DTG	M	A	19	72	TDF FTC EFV[¤]<br>(on EFV from 4w)		<20	Depression	1	no	yes	yes
**2**	**DTG**	**M**	**A**	**11**	**12**	**ABC 3TC DTG**	**20<25kg, FCT 25mg**	**<20**	**Psychosis[ψ]**	**3**	**yes**	**yes**	**yes**
**2**	**DTG**	**M**	**A**	**14**	**130**	**ABC 3TC NVP[¤]<br>(on NVP from 35w)**		**15000**	**Insomnia[ψ]**	**3**	**no**	**no**	**no**
13	DTG	F	A	16	156	ABC 3TC DTG	≥40kg, FCT 50mg	<20	Suicidal ideation	1	no	no	no
14	DTG	F	A	20	132	ABC 3TC DTG	≥40kg, FCT 50mg	<20	Suicidal ideation	1	no	no	no
15	DTG	M	A	11	52	ABC 3TC DTG	30<40kg, FCT 50mg	<20	Suicidal ideation	2	no	no	no
16	DTG	F	B	17	107	ABC 3TC DTG	≥40kg, FCT 50mg	<20	Suicidal ideation	1	no	no	no
**17**	**DTG**	**F**	**A**	**17**	**96**	**TDF 3TC DTG**	**≥40kg, FCT 50mg**	<50	**Parasuicide**	**4**	**yes**	**no**	**yes**
**17**	**DTG**	**F**	**A**	**17**	**98**	**TDF 3TC DTG**	**≥40kg, FCT 50mg**	<50	**Depression**	**4**	**yes**	**no[+]**	**yes**
18	DTG	M	B	15	48	TDF 3TC DTG	≥40kg, FCT 50mg	<20	Suicidal ideation	3	no	no	yes
19	DTG	M	A	18	145	ABC 3TC DTG	≥40kg, FCT 50mg	<40	Suicidal ideation[ψ]	1	no	no	no
20	DTG	F	A	17	93	TDF FTC DTG	≥40kg, FCT 50mg	<40	Suicide attempt	4	no	no	yes
**21**	**SOC**	**M**	**A**	**16**	**50**	**ABC 3TC EFV**		<20	**Suicidal ideation**	**2**	**no**	**no**	**yes**

**21**	**SOC**	**M**	**A**	**16**	**73**	**ABC 3TC EFV**		<20	**Suicidal ideation**	**2**	**no**	**yes**	**no**
**21**	**SOC**	**M**	**A**	**18**	**142**	**ABC 3TC ATVr (on ATVr from 73w)**		<20	**Parasuicide**	**4**	**yes**	**no**	**no**
22	SOC	M	B	18	80	ZDV 3TC LPVr		130,834	Parasuicide	4	no	no	no
**23**	**SOC**	**M**	**B**	**18**	**31**	**TDF FTC LPVr**		**525**	**Suicidal ideation**	**2**	**no**	**no**	**no**
**23**	**SOC**	**M**	**B**	**19**	**70**	**TDF FTC ATVr (on ATVr from 40w)**		**<40**	**Suicidal ideation**	**1**	**no**	**no**	**no**
**11**	**SOC**	**M**	**A**	**8**	**88**	**ABC 3TC EFV**		**56**	**Parasuicide**	**4**	**yes**	**no**	**yes**

DTG= dolutegravir. SOC=standard of care. BD=twice daily. ABC=abacavir. ATVr=ritonavir-boosted atazanavir. EFV=efavirenz. FTC=emtricitabine. LPVr=ritonavir-boosted lopinavir. TDF=tenofovir disoproxil fumarate. ZDV=zidovudine. 3TC=lamivudine. SAE=serious adverse event. DAIDS= the Division of AIDS (DAIDS) adverse events grading. Participants with multiple events are in highlighted bold font. Participants with more than one event are highlighted in bold. [§]Initial 'lower' DTG doses: 14-<30kg: 25mg FCT; ≥40 kg: 50mg FCT. Increased DTG doses (currently licensed): 14-<20kg: 25mg DT; 30-<40kg: 50mg FCT. [θ] Relatedness was adjudicated by the end-point review committee to any antiretroviral drug the patient could have received. We considered the event was related to ART if the committee concluded the event was definitely/probably or possibly related to an antiretroviral drug and the patient was receiving this drug(s) at the time of the event. [¥]Participant #1 had hypertension at the same time. [¤]Participants #2 and #12 switched off DTG before the onset of the event due to other reasons. [ψ]Three patients in the DTG group had pre-trial neuropsychiatric conditions (participant #2 with psychosis (week 12) dystonia and insomnia (week 130) had pre-trial history of abnormal behaviour, excessive talking, hyperactivity; participant #3 with convulsions had pre-trial history of bacterial meningitis and seizures; participant #19 with suicidal ideation had history of suicidal ideation in the past). One participant #10 in the SOC group, who developed seizures 2 weeks after switching to DTG, had epilepsy diagnosed pre-trial. [§]Participant #3had two SAE (two hospital admissions) for one ongoing event. *EFV dose changed from 600mg to 400mg. [Ω]Participant was on DTG 50mg BD for concomitant TB treatment with rifampicin-containing regimen.[+]Modified ART after the trial censoring date.

Table S3. Summary of participants mood-related symptoms by ODYSSEY A and ODYSSEY B

	Total			A			B		
	**DTG**	**SOC**	**Total**	**DTG**	**SOC**	**Total**	**DTG**	**SOC**	**Total**
**Participants randomised**	**350**	**357**	**707**	**154**	**157**	**311**	**196**	**200**	**396**
**Number of follow-up questionnaires [number of participants who completed ≥1 questionnaire]**	**2073 [348]**	**2011 [348]**	**4084 [696]**	**876 [153]**	**832 [151]**	**1708 [304]**	**1197 [195]**	**1179 [197]**	**2376 [392]**
**Dizziness^**									
Total # of reports over follow-up (% of all follow-up questionnaires)	92 (4%)	141 (7%)	233 (6%)	42 (5%)	77 (9%)	119 (7%)	50 (4%)	64 (5%)	114 (5%)
# of participants reporting at >=1 follow-up visit	64 (18%)	99 (28%)	163 (23%)	31 (20%)	51 (34%)	82 (27%)	33 (17%)	48 (24%)	81 (21%)
P-value*			0.001			0.008			0.069
**Low Mood^**									
Total # of reports over follow-up (% of all follow-up questionnaires)	137 (7%)	142 (7%)	279 (7%)	80 (9%)	82 (10%)	162 (9%)	57 (5%)	60 (5%)	117 (5%)
# of participants reporting at >=1 follow-up visit	83 (24%)	95 (27%)	178 (26%)	44 (29%)	52 (34%)	96 (32%)	39 (20%)	43 (22%)	82 (21%)
P-value*			0.285			0.287			0.656
**Problems Concentrating^**									
Total # of reports over follow-up (% of all follow-up questionnaires)	38 (2%)	51 (3%)	89 (2%)	25 (3%)	23 (3%)	48 (3%)	13 (1%)	28 (2%)	41 (2%)
# of participants reporting at >=1 follow-up visit	33 (9%)	41 (12%)	74 (11%)	20 (13%)	18 (12%)	38 (13%)	13 (7%)	23 (12%)	36 (9%)
P-value*			0.319			0.762			0.086
**Self-harm^**									
Total # of reports over follow-up (% of all follow-up questionnaires)	8 (0%)	1 (0%)	9 (0%)	3 (0%)	0 (0%)	3 (0%)	5 (0%)	1 (0%)	6 (0%)
# of participants reporting at >=1 follow-up visit	8 (2%)	1 (0%)	9 (1%)	3 (2%)	0 (0%)	3 (1%)	5 (3%)	1 (1%)	6 (2%)
P-value*			0.025			0.248			0.121
**Worried Often^**									
Total # of reports over follow-up (% of all follow-up questionnaires)	53 (3%)	57 (3%)	110 (3%)	28 (3%)	31 (4%)	59 (3%)	25 (2%)	26 (2%)	51 (2%)
# of participants reporting at >=1 follow-up visit	44 (13%)	46 (13%)	90 (13%)	22 (14%)	22 (15%)	44 (14%)	22 (11%)	24 (12%)	46 (12%)
P-value*			0.816			0.962			0.782

**Life Not Worth Living^**																		
Total # of reports over follow-up (% of all follow-up questionnaires)	20	(1%)	5	(0%)	25	(1%)	11	(1%)	3	(0%)	14	(1%)	9	(1%)	2	(0%)	11	(0%)
# of participants reporting at >=1 follow-up visit	17	(5%)	5	(1%)	22	(3%)	9	(6%)	3	(2%)	12	(4%)	8	(4%)	2	(1%)	10	(3%)
P-value*					0.009						0.081						0.061	
**Angry or Aggressive Often^**																		
Total # of reports over follow-up (% of all follow-up questionnaires)	140	(7%)	149	(7%)	289	(7%)	64	(7%)	67	(8%)	131	(8%)	76	(6%)	82	(7%)	158	(7%)
# of participants reporting at >=1 follow-up visit	89	(26%)	87	(25%)	176	(25%)	38	(25%)	42	(28%)	80	(26%)	51	(26%)	45	(23%)	96	(24%)
P-value*					0.864						0.555						0.446	
**Suicidal Thoughts^**																		
Total # of reports over follow-up (% of all follow-up questionnaires)	13	(1%)	0	(0%)	13	(0%)	9	(1%)	0	(0%)	9	(1%)	4	(0%)	0	(0%)	4	(0%)
# of participants reporting at >=1 follow-up visit	13	(4%)	0	(0%)	13	(2%)	9	(6%)	0	(0%)	9	(3%)	4	(2%)	0	(0%)	4	(1%)
P-value*					0.001						0.003						0.060	
**life not worth living or suicidal thoughts^**																		
Total # of reports over follow-up (% of all follow-up questionnaires)	27	(1%)	5	(0%)	32	(1%)	16	(2%)	3	(0%)	19	(1%)	11	(1%)	2	(0%)	13	(1%)
# of participants reporting at >=1 follow-up visit	23	(7%)	5	(1%)	28	(4%)	13	(8%)	3	(2%)	16	(5%)	10	(5%)	2	(1%)	12	(3%)
P-value*					0.000						0.011						0.018	

^The worst of the carer and participant reported mood symptom was taken at each study visit. * Mantel-Haenszel Chi-squared test, comparing the number of participants reporting issue at >=1 visit across treatment arms

Table S4: Reported sleep problems and overall quality of sleep by ODYSSEY A and ODYSSEY B

	Total			A			B		
	**DTG**	**SOC**	**Total**	**DTG**	**SOC**	**Total**	**DTG**	**SOC**	**Total**
**Participants randomised**	**350**	**357**	**707**	**154**	**157**	**311**	**196**	**200**	**396**
**Number of follow-up questionnaires [number of participants who completed ≥1 questionnaire]**	**2162 [349]**	**2096 [350]**	**4258 [699]**	**922 [154]**	**878 [153]**	**1800 [307]**	**1240 [195]**	**1218 [197]**	**2458 [392]**
**Time taken to sleep^**									
Total # of reports (% of all follow-up questionnaires)									
< 15 mins	1531 (71%)	1490 (71%)	3021 (71%)	617 (67%)	599 (69%)	1216 (68%)	914 (74%)	891 (74%)	1805 (74%)
>=15-30mins	496 (23%)	451 (22%)	947 (22%)	241 (26%)	200 (23%)	441 (25%)	255 (21%)	251 (21%)	506 (21%)
30-60mins	90 (4%)	85 (4%)	175 (4%)	46 (5%)	44 (5%)	90 (5%)	44 (4%)	41 (3%)	85 (3%)
>1hr	19 (1%)	31 (1%)	50 (1%)	11 (1%)	17 (2%)	28 (2%)	8 (1%)	14 (1%)	22 (1%)
don't know	20 (1%)	27 (1%)	47 (1%)	6 (1%)	14 (2%)	20 (1%)	14 (1%)	13 (1%)	27 (1%)
P-value*			0.509			0.465			0.717
# of participants reporting issue at >=1 follow-up visit+	76 (22%)	75 (21%)	151 (22%)	37 (24%)	34 (22%)	71 (23%)	39 (20%)	41 (21%)	80 (20%)
P-value**			0.914			0.708			0.842
**Nightmares and/or vivid dreams^**									
Total # of reports (% of all follow-up questionnaires)									
never	1360 (63%)	1375 (66%)	2735 (64%)	567 (61%)	550 (63%)	1117 (62%)	793 (64%)	825 (68%)	1618 (66%)
infrequently	311 (14%)	252 (12%)	563 (13%)	175 (19%)	129 (15%)	304 (17%)	136 (11%)	123 (10%)	259 (11%)
occasionally	271 (13%)	261 (12%)	532 (13%)	109 (12%)	112 (13%)	221 (12%)	162 (13%)	149 (12%)	311 (13%)
frequently	155 (7%)	143 (7%)	298 (7%)	58 (6%)	67 (8%)	125 (7%)	97 (8%)	76 (6%)	173 (7%)
don't know	63 (3%)	65 (3%)	128 (3%)	13 (1%)	20 (2%)	33 (2%)	50 (4%)	45 (4%)	95 (4%)
P-value*			0.109			0.187			0.133
# of participants reporting issue at >=1 follow-up visit+	194 (56%)	178 (51%)	372 (53%)	85 (55%)	82 (54%)	167 (54%)	109 (56%)	96 (49%)	205 (52%)
P-value**			0.210			0.778			0.156
**Sleep Quality^**									
Total # of reports (% of all follow-up questionnaires)									
very good	672 (31%)	656 (31%)	1328 (31%)	317 (34%)	306 (35%)	623 (35%)	355 (29%)	350 (29%)	705 (29%)
good	1349 (62%)	1326 (63%)	2675 (63%)	520 (56%)	507 (58%)	1027 (57%)	829 (67%)	819 (67%)	1648 (67%)
fair	104 (5%)	86 (4%)	190 (4%)	62 (7%)	48 (5%)	110 (6%)	42 (3%)	38 (3%)	80 (3%)
not that good	26 (1%)	22 (1%)	48 (1%)	16 (2%)	13 (1%)	29 (2%)	10 (1%)	9 (1%)	19 (1%)

very bad	9 (0%)	6 (0%)	15 (0%)	7 (1%)	4 (0%)	11 (1%)	2 (0%)	2 (0%)	4 (0%)
P-value*			0.894			0.249			0.963
# of participants reporting issue at >=1 follow-up visit+	30 (9%)	24 (7%)	54 (8%)	17 (11%)	12 (8%)	29 (9%)	13 (7%)	12 (6%)	25 (6%)
P-value**			0.391			0.338			0.816
**Awake in the night^**									
Total # of reports (% of all follow-up questionnaires)									
never	1738 (80%)	1724 (82%)	3462 (81%)	683 (74%)	691 (79%)	1374 (76%)	1055 (85%)	1033 (85%)	2088 (85%)
infrequently	218 (10%)	171 (8%)	389 (9%)	126 (14%)	89 (10%)	215 (12%)	92 (7%)	82 (7%)	174 (7%)
occasionally	106 (5%)	96 (5%)	202 (5%)	52 (6%)	45 (5%)	97 (5%)	54 (4%)	51 (4%)	105 (4%)
frequently	88 (4%)	86 (4%)	174 (4%)	57 (6%)	45 (5%)	102 (6%)	31 (3%)	41 (3%)	72 (3%)
don't know	10 (0%)	19 (1%)	29 (1%)	4 (0%)	8 (1%)	12 (1%)	6 (0%)	11 (1%)	17 (1%)
P-value*			0.748			0.152			0.940
# of participants reporting issue at >=1 follow-up visit+	126 (36%)	131 (37%)	257 (37%)	67 (44%)	64 (42%)	131 (43%)	59 (30%)	67 (34%)	126 (32%)
P-value**			0.706			0.767			0.426
**Trouble staying awake^**									
Total # of reports (% of all follow-up questionnaires)									
never	1841 (85%)	1753 (84%)	3594 (85%)	757 (82%)	706 (81%)	1463 (81%)	1084 (88%)	1047 (86%)	2131 (87%)
infrequently	142 (7%)	147 (7%)	289 (7%)	76 (8%)	78 (9%)	154 (9%)	66 (5%)	69 (6%)	135 (6%)
occassionally	90 (4%)	97 (5%)	187 (4%)	43 (5%)	39 (4%)	82 (5%)	47 (4%)	58 (5%)	105 (4%)
frequently	79 (4%)	81 (4%)	160 (4%)	41 (4%)	46 (5%)	87 (5%)	38 (3%)	35 (3%)	73 (3%)
don't know	7 (0%)	16 (1%)	23 (1%)	5 (1%)	8 (1%)	13 (1%)	2 (0%)	8 (1%)	10 (0%)
P-value*			0.491			0.398			0.077
# of participants reporting issue at >=1 follow-up visit+	105 (30%)	111 (32%)	216 (31%)	50 (32%)	55 (36%)	105 (34%)	55 (28%)	56 (28%)	111 (28%)
P-value**			0.636			0.520			0.961

^The worst of the carer and participant reported sleep was taken at each study visit. +Issue defined as: >=30 mins to get to sleep; occasionally or frequently being awake in the night, trouble staying awake during the days, or having nightmares and/or vivid dreams; and sleep quality being 'not that good' or 'very bad'. * Ordered logistic mixed models are used to compare outcomes across treatment arms over time with a random effect for intercept and fixed effects for treatment arm, study visits and adjustment stratification factors. ** Mantel-Haenszel Chi-squared test, comparing the number of participants reporting issue at >=1 visit across treatment arms.

ODYSSEY (PENTA 20)

A randomised trial of dolutegravir (DTG)-based antiretroviral therapy vs. standard of care (SOC) in children with HIV infection starting first-line or switching to second-line ART (PENTA 20)

ISRCTN #: ISRCTN91737921
EUDRACT #: 2014-002632-14
NCT #: NCT02259127

**Version Number and Date:** 6.0 17 June 2021<br>**Supersedes version:** 5.1 16 June 2021			
**Author**	**Position**	**Initial**	**Date**
Dr Ellen White	Delegated Statistician		
**Approved by**			
Dr Deborah Ford	Trial Statistician		
Professor Diana Gibb	Chief Investigator		

**Version**	**Date**	**Who**	**Comments**
0.1	6March2016	Angela Crook<br>Aimee Nunn	Protocol Version 2.0
0.2	15June2016	Cecilia Moore	First Draft
0.3	08August2016	Cecilia Moore	Second Draft, in response to A. Babiker and D. Ford's comments.
0.4	12Jan2017	Cecilia Moore	Third Draft, on the basis of amendments made to the protocol
0.5	06Mar2017	Cecilia Moore	Fourth Draft, on the basis of comments from D. Ford
0.6	16Mar2017	Cecilia Moore	Fifth Draft, on the basis of comments from A. Babiker
0.7	20Mar2017	Cecilia Moore	Sixth Draft, on the basis of comments from D. Gibb
0.8	03Apr2017	Cecilia Moore	Seventh Draft, on the basis of comments from A. Turkova
1.0	05Apr2017	Cecilia Moore	Converted to final version for circulation
1.1	28Oct2018	Cecilia Moore	Update on basis of Protocol version 4.0 release<br>• Inclusion of children 3-<14kg with randomisation stratified by weight band and ODYSSEY A/B.<br>Changes made to the definition of one component of the primary endpoint<br>• Failure defined by <1 log_{10} drop at week 24 modified to failure for <1 log_{10} drop at week 24 <u>and</u> switch to second/third line ART for treatment failure. Approved by IDMC (19/02/2018); see meeting documents.

			Methods clarified with respect to missing VL data at week 24<br>Modification of definition of ART modifying adverse event<br>Corrected baseline viral load calculation<br>Changes made to presentation of lipid changes based on feedback from second IDMC meeting (19/02/2018)<br>• Add total cholesterol/HDL ratio and non-HDL<br>• Add presentation of A/B separately if indication of interaction effect<br>Modification made to the calculation of virological failure time and censoring time for primary endpoint.<br>Addition of sensitivity analysis for the primary endpoint.<br>Addition of analysis of sleep and mood questionnaires<br>Modification to method of analysis of VL <50 c/ml and VL <400 c/ml at cross-sectional time points
1.2	22Nov2018	Debbie Ford	Clarifications made following review by ViiV<br>Naomi Givens – Director, Statistics<br>Ralph DeMasi – Head of Statistics<br>Judy Hopking – Principal Statistician
2.0	03Dec2018	Cecilia Moore	Converted to final version for circulation
2.1	30Jan2019	Cecilia Moore	Modification to the presentation of results if indication of interaction effect following review by IDMC members at the third IDMC meeting (14Dec2018)<br>• Results in A/B and Total population will be presented irrespective of interaction effect
2.2	10June2019	Becky Turner	Bayesian approach for analysis of children <14kg at randomisation added
3.0	14November2019	Debbie Ford	Clarifications added around testing multiple outcomes. Reviewed by Abdel Babiker.
3.1	17September2020	Debbie Ford/Ellen White	Reviewed for changes needed due to extended follow-up in Thailand/Africa post randomised phase<br>Modified to allow for end of randomised phase visits taking place outside the original window<br>Clarification around resistance analysis<br>Added COVID-19 as a notable event
4.0	29 September 2020	Ellen White	Converted to final version for circulation
4.1	18 January 2021	Ellen White	Clarification around adverse event analysis<br>Clarification around sensitivity analyses<br>Clarification around grade 3/4 AEs which include all AEs grade ≥3
4.2	19 January 2021	Ellen White	Updated based on Debbie Ford's comments
5.0	19 January 2021	Ellen White	Converted to final version for circulation
5.1	13 June 2021	Debbie Ford	Changes made to description of analysis of cohort enrolled <14kg.<br>Due to COVID, children have all remained in randomised follow-up to 28 June 2021, as opposed to exiting at 96 weeks, hence analysis will follow the same approach as in ≥14kg with an end of trial censoring date.<br>Following discussions with Ian White, agreed to estimate CIs for probability of failure on log(-log(S(t)) scale in cohort <14kg due to low numbers of children (section 6.6).<br>We also clarified the use of the bias corrected bootstrap for the CIs for the probability of failure in the main trial cohort and for the risk difference in the main trial cohort and the <14kg cohort.
6.0	17 June 2021	Debbie Ford	Accepted a correction in section 6.6 following review by Ian White and converted to final version.

1. BACKGROUND AND DESIGN 16
1.1 BACKGROUND AND AIMS 16
1.1.1 *Hypothesis* *16*
1.2 TRIAL DESIGN 16
FIGURE 1. TRIAL SCHEMA 17
1.3 PATIENT SELECTION 18
1.3 TRIAL INTERVENTIONS 18
1.4 DURATION OF TRIAL 19
1.5 METHOD OF RANDOMISATION 19
1.6 SAMPLE SIZE CALCULATIONS 20
2. OUTCOME MEASURES 20
2.1 PRIMARY EFFICACY OUTCOME 20
2.2 SECONDARY EFFICACY OUTCOMES 20
2.3 SECONDARY SAFETY OUTCOMES 20
2.4 OTHER SECONDARY OUTCOMES 20
3. ANALYSIS POPULATIONS 21
4. DERRIVATION OF DATA TO BE ANALYSED 21
4.1 DEFINITION OF BASELINE 21
4.2 DEFINITION OF FOLLOW-UP TIME 22
4.3 DEFINITION OF NOMINAL WEEK FOR LABORATORY MEASUREMENT AND OTHER CLINICAL PARAMETERS 22
4.4 DEFINITION OF TREATMENT CHANGES 22
4.5 HANDLING OF VIRAL LOAD DATA 23
4.6 TRIAL CENSORING DATE AND ENDPOINT DETERMINATION 24
A) PARTICIPANTS ≥14KG 24
4.7 STRATIFICATION VARIABLES 24
4.8 DEFINITION OF NEW RESISTANCE MUTATIONS 25
4.9 HANDLING ADVERSE EVENTS 25
5. ANALYSIS PRINCIPLES 25
6. ANALYSIS DETAILS 26
6.1 ENROLMENT AND ELIGIBILITY 27
6.2 BASELINE CHARACTERISTICS 27
6.3 FOLLOW-UP 28
6.4 ANTIRETROVIRAL THERAPY RECEIVED 28
6.5 AVAILABILITY OF HIV-1 RNA MEASUREMENTS 29
6.6 PRIMARY EFFICACY OUTCOME 29
6.6.1 *Sensitivity analyses for the primary endpoint* *29*
6.6.2 *Subgroup analyses for the primary endpoint* *30*
6.7 SECONDARY EFFICACY ANALYSES 30
6.8 SECONDARY SAFETY OUTCOMES 31
6.9 OTHER SECONDARY OUTCOMES 32
6.10 OTHER OUTCOMES 32
6.11 TESTING MULTIPLE OUTCOMES 33
7. SUBGROUP ANALYSES 33
7.1 ANALYSES BY DOSE AND WEIGHT-BAND 33
8. ANCILLARY STUDIES AND SUBSTUDIES 33
APPENDIX I 35

BACKGROUND AND DESIGN

1.1 Background and Aims

With the development of new antiretroviral drugs (ARVs), including new drug classes, which offer simplified, more potent options for first- and second-line therapy with fewer adverse effects in adults, there is a need to evaluate their place in paediatric HIV treatment.

Unique issues for HIV-infected children include: potential for perinatal transmission of viruses resistant to antiretroviral therapy (ART) regimens taken by mothers during pregnancy and breastfeeding; special consideration of adverse events as children take drugs during growth and development and will be on them for much longer than adults; and the need for age appropriate once daily formulations, preferably fixed dose scored dispersible combinations which may require different ratios of drugs from those used in adults.

Globally, more than 80% of HIV-infected children live in Sub Saharan Africa (SSA). Although there has been substantial improvement in ART coverage over the last few years, only half of children in need of treatment have access to ART (1, 2). Rapid disease progression and high early mortality require that ART is started early in life and many children still die before they are diagnosed. Children <3 years in low/middle income countries are taking either non-nucleoside reverse transcriptase inhibitors (NNRTI)-based ART (e.g. most African countries, India, Eastern Europe) or ritonavir boosted protease inhibitor (bPI) lopinavir/ritonavir (LPV/r)-based ART (South Africa; some centres of excellence in SSA). Children >3 years in SSA, including South Africa, start NNRTI-based ART. In both well-resourced and resource-limited settings, there is a need for alternative first- and second-line regimens which can be given in the face of possible transmitted resistance (from prevention of mother to child transmission pMTCT) or resistance arising from first-line ART, whether PI or NNRTI based. New regimens need to be potent, durable, non-toxic and available in acceptable once daily formulations with correct drug ratios. Ideally regimens should be robust with a high barrier to resistance, have minimal cross-resistance and minimal interactions with anti-tuberculosis drugs.

Dolutegravir (DTG) is a second-generation integrase inhibitor (INSTI) with the advantage of once daily dosing, a good short-term safety profile, low pharmacokinetic variability, few drug-to-drug interactions, rapid and robust virological response with an optimised background treatment, a distinct resistance profile from raltegravir, a high genetic barrier to resistance and high potency at a low milligram dose. Combination regimens with DTG will therefore be attractive for children starting first-line ART and for those switching to second-line for reasons of toxicity or failure.

ODYSSEY will be a pragmatic strategy trial evaluating the efficacy and safety of once daily DTG-based ART compared with standard of care in children and adolescents starting first- or second-line ART in resource-limited and well-resourced settings.

1.1.1 Hypothesis

DTG + 2 NRTIs is non-inferior to SOC (NNRTI or PI or INSTI + 2 or 3 NRTIs) in terms of efficacy and superior in terms of toxicity profile.

1.2 Trial design

ODYSSEY is an open-label, multi-centre, randomised (1:1), non-inferiority, Phase II/III, 96-week, 2-arm clinical trial.

Figure 1. Trial Schema

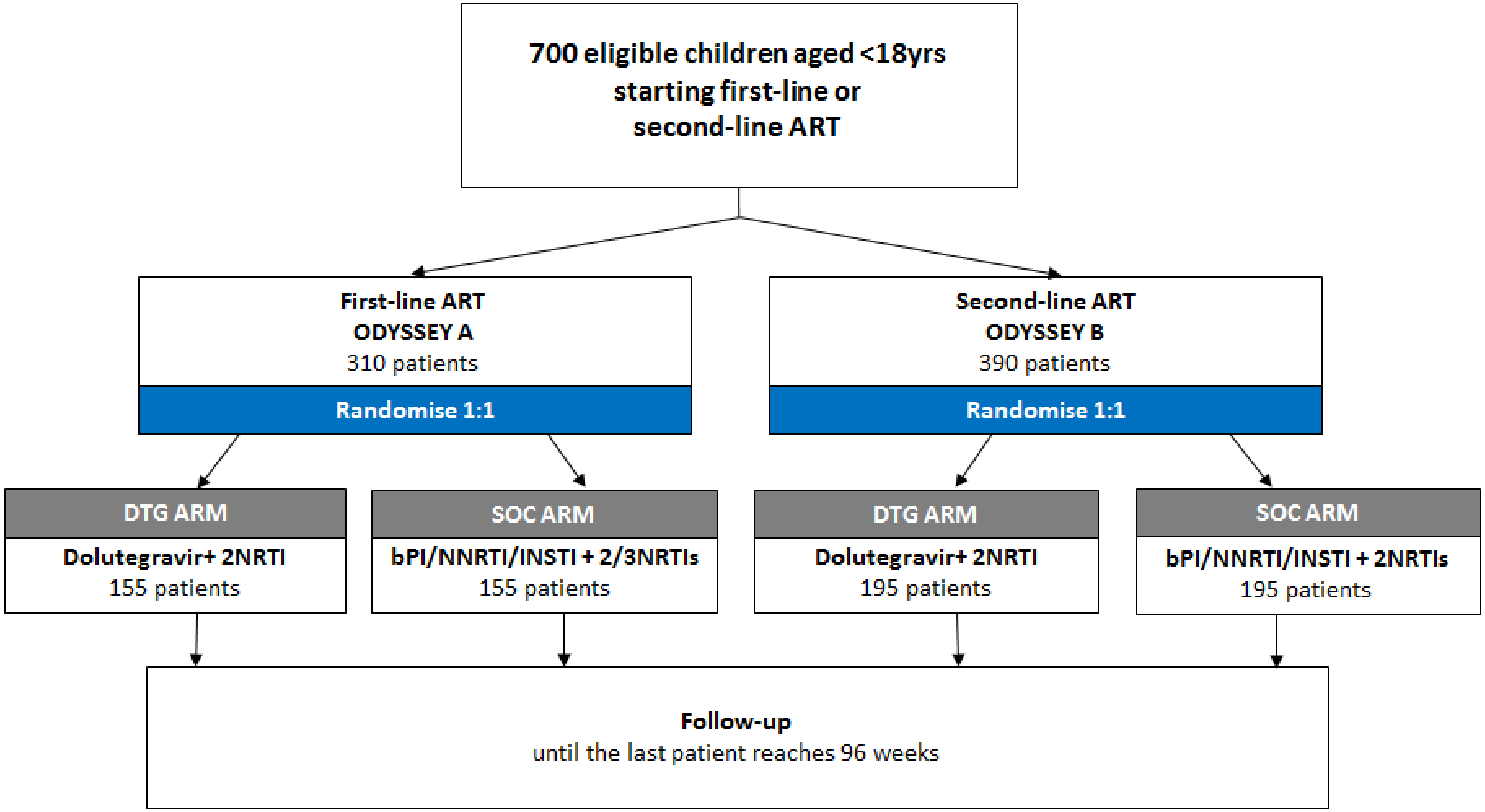

Note:
At least 60 additional children weighing 3-<14kg will be enrolled and followed up for 96 weeks

1.3 Patient selection

Eligible participants are HIV-1 infected children younger than 18 years planning to start first-line or second-line antiretroviral therapy. Additional inclusion criteria are as follows:

- Children ≥28 days and <18 years weighing ≥3kg with confirmed HIV-1 infection*
- Parents/carers and children, where applicable, give informed written consent
- Girls who have reached menses must have a negative pregnancy test at screening and randomisation and be willing to adhere to effective methods of contraception if sexually active
- Children with co-infections who need to start ART according to local/national guidelines
- Parents/carers and children, where applicable, willing to adhere to a minimum of 96 weeks' follow-up

*Recruitment opened in children ≥6 years and <18 years weighing ≥20kg (Protocol version 2.0). In February 2017 the EMA approved DTG for children ≥6 years down to 15kg (with a 20mg QD dose for children weighing 15-<20kg). Following this, in May 2017, ODYSSEY opened for recruitment of these children across all sites (Version 2.0). Version 3.0 included recruitment of children ≥14kg (with no age restriction) with a lead-in PK sub-study for children weighing 14 to <20kg. Version 4.0 allows for recruitment of children ≥3kg and ≥28 days; children weighing 3 to <14kg must be willing to participate in a lead-in PK substudy to enter the trial. Throughout the trial eligibility will be based in both arms on availability of DTG dose given age/weight band.

Additionally for ODYSSEY A, participants must be planning to start first-line ART.

Additionally for ODYSSEY B, participants must be:

- Planning to start second-line ART defined as either: (i) switch of at least 2 ART drugs due to treatment failure; or (ii) switch of only the third agent due to treatment failure where drug sensitivity tests show no mutations conferring NRTI resistance
- Treated with only one previous ART regimen. Single drug substitutions for toxicity, simplification, changes in national guidelines or drug availability are allowed
- At least one NRTI with predicted preserved activity available for a background regimen
- In settings where resistance tests are routinely available, at least one active NRTI from TDF/TAF, ABC or ZDV should have preserved activity based on cumulative results of resistance tests
- In settings where resistance tests are not routinely available, children who are due to switch according to national guidelines should have at least one new NRTI predicted to be available from TDF/TAF, ABC or ZDV
- Viral load ≥500 c/ml* at screening visit or within 4 weeks prior to screening

* Protocol version 3.0 onwards (viral load ≥1000 c/ml in protocol version 2.0)

Exclusion criteria are as follows:

- History or presence of known allergy or contraindications to DTG
- History or presence of known allergy or contraindications to proposed available NRTI backbone or proposed available SOC third agent.
- Alanine aminotransferase (ALT) ≥5 times the upper limit of normal (ULN) or ALT ≥3xULN and bilirubin ≥2xULN
- Patients with severe hepatic impairment or unstable liver disease (as defined by the presence of ascites, encephalopathy, coagulopathy, hypoalbuminaemia, oesophageal or gastric varices, or persistent jaundice), known biliary abnormalities (with the exception of Gilbert's syndrome or asymptomatic gallstones)
- Anticipated need for Hepatitis C virus (HCV) therapy during the study
- Pregnancy or breastfeeding
- Evidence of lack of susceptibility to integrase inhibitors or more than a 2-week exposure to antiretrovirals of this class

1.3 Trial interventions

Children starting first- and second-line ART will be randomised separately (both in 1:1 ratio):
ODYSSEY A (first-line ART):

1. DTG + 2 NRTIs
2. SOC (defined as PI, NNRTI or INSTI+ 2 or 3 NRTIs)

ODYSSEY B (second-line ART):

1. DTG + 2 NRTI

2. SOC (defined as PI, NNRTI or INSTI + 2 NRTIs)

1.4 Duration of trial

A minimum of 700 children will be enrolled over 96-120 weeks. This will include ≥310 children starting first-line (ODYSSEY A) and ≥390 children starting second-line ART (ODYSSEY B). After this target is reached the end of main trial recruitment date will be defined. Recruitment will then stop for children ≥14kg. Recruitment will continue in the 3 lower weight bands (3-<6kg, 6-<10kg and 10-<14kg) until at least 20 children are recruited in each weight band. Follow-up for children recruited up to the end of main trial recruitment date will continue until the last child recruited in the main trial has completed 96 weeks follow-up (herein referred to as the end of the main trial, 24th April 2020); each participant in the main trial will then have a final trial visit. Analysis of the main trial will be conducted at this time.

Each of the children recruited in the lower weight bands (<14kg) after the end of main trial recruitment date will continue to be followed up for 96 weeks (from their date of randomisation); we will ask sites to schedule their 96 week visit for on/after 96 weeks and the trial will remain open until this follow-up is complete.

Note: in practice there was no overlap of recruitment of children ≥14kg (n=707) and recruitment of children <14kg (N=85) hence children randomised ≥14kg to 22/June/2018 constitute the main trial population.

Protocol version 6.0 allows for extended follow-up in African and Thai sites of ODYSSEY participants post end of randomised follow-up; European sites are not participating in extended follow-up. For each participant their end of randomised phase visit can be identified by a question on the follow-up form.

Due to the COVID-19 pandemic (March 2020 onwards), and uncertainty about how it would evolve and ability of clinics to conduct face-to-face visits, sites were advised they could conduct end of randomised phase visits for the main trial outside of the pre-defined window (24th April 2020 (96 weeks from date last participant recruited ≥14kg) –30 June 2020) for participants who had completed 96 weeks follow-up. This resulted in one child being seen early in Europe and exiting from the trial on 6th April 2020 before the last participant reached 96 weeks (trial censoring date_24th April 2020). In Thailand and Africa, many participants were kept in follow-up after 30 June 2020 to ensure continued drug supply and until a face-to-face visit could be done; sites were subsequently asked to complete end of randomised phase visits for participants recruited weighing ≥14kg by end November 2020.

To ensure continued care and drug supply for children recruited <14kg (who were not due to reach 96 weeks prior to May 2020 at the earliest), sites were advised to retain all children <14kg in the randomised phase of the trial until advised otherwise. Subsequently it was decided to retain children recruited <14kg in randomised follow-up until the last child recruited in this cohort reaches 96 weeks (28 June 2021), with each child having an end of randomised phase visit at their next scheduled visit (June-September 2021).

During COVID-19, phone visits were conducted for some participants; this information was captured and sites were asked to ensure the week 96 and the end of randomised phase visit were in person.

1.5 Method of Randomisation

Randomisation of children weighing ≥14kg will be stratified by:

- Antiretroviral treatment: naïve or experienced (ODYSSEY A or ODYSSEY B)
  - Routine availability at site of resistance tests vs. non availability of resistance tests, for children failing treatment
  - bPI vs. non-bPI ART offered as SOC*
  - NRTI backbone* : ABC/3TC or ABC/3TC/ZDV vs. TDF/3TC or TDF/FTC or TAF/3TC or TAF/FTC vs. Other

Randomisation of children weighing 3-<14kg will be performed separately stratified by

- antiretroviral treatment: naïve or experienced (ODYSSEY A or ODYSSEY B)
- weight band at randomisation: 3-<6kg vs. 6-<10kg vs. 10-<14kg

Randomisation lists will be generated using permuted blocks of varying size.

* chosen prior to randomisation

1.6 Sample size calculations

Based on previous HIV studies a non-inferiority margin (delta) of 10-12% is considered acceptable[1,2]. Assuming a failure rate of 18% overall (both first-line and second-line children combined) in the DTG arm and SOC arm by 96 weeks and allowing for 10% loss to follow up, 700 children will provide 90% power to exclude (at two-sided 5% significance level, or equivalently at one-sided 2.5% significance level) a difference of more than 10% between the two arms. If the upper bound of the two-sided 95% confidence limit for the difference in the proportion failing (DTG – SOC) is less than 10% (the margin of non-inferiority) the DTG-regimen will be considered to be non-inferior to the SOC.

Enrolling 310 first-line ART and 390 second-line ART children will provide 80% power to exclude a difference of more than 12% between the DTG and SOC arms in both subgroups, assuming a failure rate of 15% in ART-naïve (based on previous trials, including the recent ARROW trial (36) in Africa), and 20% in ART-experienced with loss to follow up of 10%.

3. OUTCOME MEASURES

2.1 Primary efficacy outcome

The primary outcome is the difference in the cumulative probability of virological or clinical failure by 96 weeks, estimated by Kaplan-Meier methods using time to the first occurrence of any of the following components:

- Insufficient virological response defined as <1 log10 drop at week 24 (or not <50 at week 24 if VL<500 at baseline) and switch to second/third-line treatment for treatment failure
- VL ≥400 c/ml at or after 36 weeks confirmed by next visit
- Death due to any cause
- Any new or recurrent AIDS defining event (WHO 4) or severe WHO 3 event*, confirmed by the Endpoint Review Committee

The primary comparison for the study is to compare DTG-based regimen versus SOC for the primary efficacy outcome in the combined population (naïve and experienced) adjusted for stratification variables; subpopulation analyses (ODYSSEY A and ODYSSEY B) are key secondary comparisons.

* For definitions of AIDS defining event (WHO 4) and severe WHO 3 events for the ODYSSEY trial see Appendix I

2.2 Secondary efficacy outcomes

- Proportion with clinical or virological failure (as defined above) by 48 weeks
- Time to any new or recurrent AIDS defining event (WHO 4) or severe WHO 3 event, confirmed by the Endpoint Review Committee
- Proportion with VL <50 c/ml at 48 and 96 weeks
- Proportion with VL <400 c/ml at 48 and 96 weeks
- Rate of clinical events over 96 weeks: WHO 4, severe WHO 3 events and death
- Change in CD4 count (actual values and percentage) and CD4/CD8 ratio from baseline to weeks 48 and 96
- Proportion developing new resistance mutations

2.3 Secondary safety outcomes

- Change in total cholesterol, triglycerides and lipid fractions (LDL, HDL) from baseline to weeks 48 and 96
- Incidence of serious adverse events
- Incidence of new clinical and laboratory grade 3 and 4 adverse events*
- Incidence of adverse events (of any grade) leading to treatment modification

*In ODYSSEY, deaths were coded as grade 4 events.

2.4 Other secondary outcomes

- Quality of life
- Adherence and acceptability

4. ANALYSIS POPULATIONS

Intent-to-treat population

The intent-to-treat population will include all randomised patients, except those considered randomised in error. Randomisation in error will be judged by the participant meeting a major violation of the eligibility criteria including the patient was HIV negative or the patient was randomised at a time when the dolutegravir dose was not known for the patient's weight (and patient not participating in PK); and will not depend on treatment allocation or post-randomisation follow-up.

Per protocol population

Participants who did not meet the eligibility criteria or where there was a major protocol deviation, e.g. site prescribed incorrect drug, will also be excluded from this population. Changes to the third agent (i.e. the non-NRTI component, including adding an additional third agent) for toxicity, failure or pregnancy will be defined as a change in therapy and participants will be censored at this date. Any stop in regimen for >31 days will be defined as stopping therapy and participants will be censored at this date.

Intent-to-treat analyses will be performed on all patients in the ITT population and patients will be analysed according to the study group to which they were randomised.

The primary analysis will include participants randomised prior to the end of main trial recruitment date; these include participants weighing ≥14kg at enrolment. This analysis will take place when the last of these children reaches 96 weeks follow-up and all children have had a face-to-face visit on/after 24th April 2020.

A sensitivity analysis of the primary endpoint will be done in the per protocol population (as above) by: censoring follow-up after a participant changes their third agent (i.e. non-NRTI component) (including adding an additional third agent) in their initial trial regimen for toxicity, failure, protocol deviations or pregnancy; and excluding participants who did not meet the eligibility criteria

There will be a secondary analysis of primary and secondary endpoints in all children recruited weighing<14kg (where DTG dose is determined) when this group has completed randomised follow-up. The primary endpoint will also be re-evaluated in all children including those in the main trial and children recruited <14kg (where DTG dose is determined) when follow-up in everyone to a minimum of 96 weeks is complete.

5. DERRIVATION OF DATA TO BE ANALYSED

4.1 Definition of Baseline

Baseline is defined as the date of randomisation.

For all variables, the day 0 measurement is defined as the latest measurement up to 42 days prior to (and including) date of randomisation. If no such measure exists, we will use the earliest measurement after randomisation but only up to and including 7 days after randomisation and before initiation of trial intervention.

Baseline measures of CD4, CD4 percentage, CD8, CD8 percentage and total lymphocytes will be presented as the average of all measurements taken between -42 days and day 0.

For HIV RNA, we will use log10 transformation and take the mean of the $\log_{10}$ measurements between -42 days and day 0 if available. In order to take into account measurements that are above or below the limits of detection, we will use the following:

First VL measurement	Second VL measurement	Imputed baseline $\log_{10}$ VL*
<X	<Y	If X~=Y: Minimum of $\log_{10}(X)$ and $\log_{10}(Y)$<br>If X=Y: $\log_{10}(X)$
<X	=Y	$(\log_{10}(X)+\log_{10}(Y))/2$

=X	<Y	$(\log_{10}(X)+\log_{10}(Y))/2$
=X	=Y	$(\log_{10}(X)+\log_{10}(Y))/2$

*X and Y are absolute viral load measurements between -42 days and day 0

If more than two viral loads are available between -42 days and day 0 a similar approach will be used. The anti-log of the mean log of viral loads will be taken to get absolute viral load at baseline. Baseline values for all other laboratory measures will be presented as the day 0 measurement (as defined above).

4.2 Definition of Follow-up Time

Time will be measured from randomisation. Analyses will include all follow-up until the end of the main trial or 96 weeks as appropriate [for interim analyses the date of data freeze will be used]. For analyses by scheduled visits, only time-points where there are at least 10 participants in each group with a measurement will be included.

4.3 Definition of Nominal Week for Laboratory Measurement and other clinical parameters

Laboratory measurements, and other clinical parameters (e.g. weight), at any nominal week are defined using exact dates as those taken nearest to the nominal week within equally spaced windows according to the protocol visit schedule (note that the visit schedule changes frequency). The midpoint between two scheduled visit weeks should be taken as belonging to the latter window (see below). Where there are two values within one of these equally spaced windows, but both equidistant from the nominal visit week, the later value will be used.

Week 4: 2≤ week <8
Week 12: 8≤ week <18
Week 24: 18≤ week <30
Week 36: 30≤ week <42
Week 48: 42≤ week <54 etc.

4.4 Definition of Treatment Changes

A strict initial regimen will be defined as strictly the regimen allocated at trial entry.

A substituted initial regimen will be defined as the strict initial regimen allowing for substitutions to the third agent of the regimen for reasons other than toxicity, treatment failure, pregnancy or major protocol deviation (e.g. site prescribing incorrect drug) or to the NRTI component for any reason. Permissible substitutions to the third agent include for reasons of simplification, drug availability, patient/carer decision and increases in weight.

All reductions to NRTIs are also considered substituted initial regimen.

All changes to the third agent not defined above as a substitution to the initial regimen will be considered a change from the initial treatment regimen. In the DTG arm, DTG should only be stopped for toxicity, failure or (at clinician's discretion) pregnancy.

Changes to the third agent for reasons of treatment failure will be defined as switching to second line therapy in ODYSSEY A or third line therapy in ODYSSEY B.

Any interruptions or reductions to a regimen for less than or equal to 31 consecutive days are allowed; as long as the total number of drugs allocated in the regimen is not increased.

A stop to the initial regimen will occur when a patient interrupts their regimen or their third agent for >31 days.

For the definition of adverse events leading to modification of ART, any reduction or substitution in therapy will be considered a modification of ART. Interruptions to therapy where the same drugs are subsequently restarted will not be considered a modification of ART.
We will also report adverse events leading to increased doses of ART due to concomitant medications (e.g. DTG increase for TB treatment). We will also report pregnancies leading to ART modification.

4.5 Handling of Viral Load Data

Participants are included in ODYSSEY both from sites where HIV RNA is routinely measured at site and from sites where HIV RNA is not routinely measured. In the latter case, HIV RNA will be measured retrospectively on plasma samples stored at each clinic visit. Confirmatory HIV RNA measures are likely to be performed faster in sites where HIV RNA monitoring occurs routinely. The primary endpoint includes confirmed HIV RNA≥400 c/ml at or after 36 weeks confirmed by the next visit.

For consistency across sites, where 2 consecutive HIV RNA are ≥400c/ml the date of the next scheduled visit following the first HIV RNA ≥400 (using schedule from randomisation) will be used for calculating the time to the first confirmed viral load measurement ≥400c/ml (primary endpoint failure date), rather than the date of the confirmatory measure. Where a patient has an HIV RNA≥400c/ml and switches treatment for treatment failure without a confirmatory viral load before switch, the date of the next scheduled visit following the first HIV RNA ≥400c/ml will be used for the first confirmed viral load measurement ≥400c/ml. This should be uncommon as extra plasma is taken if an unscheduled visit is made at the point of treatment failure. Treatment switch in itself is not a component of the primary endpoint; thus any patient who switches treatment for immunological/clinical failure without reaching the primary endpoint (with no HIV RNA ≥400c/ml) will not be considered a failure at time of switch.

If a HIV RNA is missing at or after 36 weeks prior to final visit this will be assumed to be <400 if the next available viral load is <400. The table below illustrates how the primary outcome will be counted when missing visits occur.

HIV RNA before missing visit(s) (copies/ml)	HIV RNA after missing visit(s) (copies/ml)	Date of virological failure	Failure status
<400	<400	Not applicable. Assume virally suppressed between visits.	Virally suppressed
≥400 but not confirmed*	≥400*	Date of next scheduled visit (using schedule from randomisation) following first HIV RNA≥400	Viral Failure
	<400	Not applicable. Assume virally suppressed between visits	Virally suppressed

* Treatment switch for failure at or after an unconfirmed HIV RNA ≥ 400 c/ml will be considered as confirmation of viral failure

The primary endpoint includes insufficient virological response by week 24, defined by less than a 1 log10 drop in viral load from baseline (or HIV RNA≥50 at week 24 when baseline HIV RNA<500) with switch to second/third line treatment for treatment failure. A participant will be considered a failure if they switch to second or third line treatment following insufficient virological response at week 24 without suppressing (VL<50) between week 24 and switching therapy. Date of switching therapy will be used as the primary endpoint failure date.

If HIV RNA is missing at week 24, we will use HIV RNA measured at week 36. Participants with no baseline viral load will be treated as meeting this endpoint if their HIV RNA ≥1000 at week 24 (week 36, if missing at 24) and the participant switches to second/third line without suppressing first.

If a participant does not attend visits at weeks 24 and 36, they will be censored at their last visit prior to week 24 (for analysis of the primary endpoint). If a participant has attended week 24 and week 36 visits but has no viral load data at either week and does not switch to second or third line treatment they will not be treated as meeting this endpoint and will not be censored for analysis of the primary endpoint. We expect few/no occurrences of missing HIV RNA at both weeks 24 and 36 (if participants are attending visits) as missing HIV RNA at week 24 will be flagged in real time with sites.

For the cross-sectional analysis at weeks 48 and 96, only participants with non-missing VL data will be included

4.6 Trial Censoring Date and Endpoint Determination

a) Participants ≥14kg

Follow-up for children ≥14kg will continue until the last child recruited has completed 96 weeks follow-up; the main trial censoring date is 24 April 2020. A participant will be considered lost to follow-up if he/she has not reached the primary endpoint (clinical or virological failure) and an HIV-RNA measurement is not available at/after 24th April 2020; in this case, follow-up will be censored at the date of last HIV RNA for assessment of combined clinical and virological failure. Participants who attended their end of randomised phase early (prior to 24 April 2020) due to COVID-19 will be described and their follow-up censored at their end of randomised phase visit.

If a participant has both virological and clinical failure, the earliest failure date will be taken as the failure date. If an HIV-RNA measurement is available at the end of randomised phase visit and the participant has not reached the primary endpoint prior to or at the time of the trial censoring date then they will be censored at the trial censoring date.

b) Participants <14kg

Although follow-up for children <14kg was originally to 96 weeks for each individual child, children were retained in randomised follow-up due to COVID-19. Therefore, follow-up for the children <14kg will continue until last child recruited reaches 96 weeks; the censoring date for this cohort is 28 June 2021. A participant will be considered lost to follow-up if he/she has not reached the primary endpoint (clinical or virological failure) and an HIV-RNA measurement is not available at/after 28 June 2021; in this case follow-up will be censored at the date of last HIV RNA for assessment of combined clinical and virological failure. If a participant has both virological and clinical failure, the earliest failure date will be taken as the failure date. If an HIV-RNA measurement is available at the end of randomised phase visit and the participant has not reached the primary endpoint by the censoring date for this cohort then they will be censored at 28 June 2021.

4.7 Stratification Variables

Where analyses of ≥14kg participants adjust for stratification variables these will include

- antiretroviral treatment [naïve or experienced (ODYSSEY A or ODYSSEY B)]
- routine availability at site of resistance tests vs. non availability of resistance tests for children failing treatment
- pre-specified NRTI backbone [ABC+3TC (± ZDV) vs. TDF(or TAF)+3TC(or FTC) vs. other]

It is likely that we will have few patients falling within the 'other' NRTI backbone stratum; in this circumstance, we will combine this stratum with the TDF (or TAF) + 3TC(or FTC) stratum.

Although randomisation is stratified by pre-specified use of bPI-based ART vs. other ART as SOC, this is likely to be strongly confounded by ODYSSEY A and B stratification; in this circumstance we will adjust for ODYSSEY A vs. ODYSSEY B and not for pre-specified use of bPI-based ART vs. other ART as SOC.

Where analyses of <14kg participants adjust for stratification variables these will include antiretroviral treatment [naïve or experienced (ODYSSEY A or ODYSSEY B)], provided sufficient numbers allow for this.

Where analysis of all participants (≥14kg+<14kg) adjust for stratification factors the primary analysis will include weight band (≥/<14kg) in addition to the strata identified above for the ≥14kg group and antiretroviral treatment (ODYSSEY A/B) in the <14kg group, provided sufficient numbers allow for this
(e.g. 10 stratums:
≥14kg/ODYSSEY A/No Resistance Testing/ABC+3TC+ZDV;
≥14kg/ODYSSEY A/Resistance Testing/ABC+3TC+ZDV;
≥14kg/ODYSSEY A/No Resistance Testing/TDF/TAF+3TC/FTC or Other;
≥14kg/ODYSSEY A/Resistance Testing/ TDF/TAF+3TC/FTC or Other;
≥14kg/ODYSSEY B/No Resistance Testing/ABC+3TC+ZDV;
≥14kg/ODYSSEY B/Resistance Testing/ABC+3TC+ZDV;
≥14kg/ODYSSEY B/No Resistance Testing/TDF/TAF+3TC/FTC or Other;
≥14kg/ODYSSEY B/Resistance Testing/ TDF/TAF+3TC/FTC or Other;
<14kg/ODYSSEY A;

<14kg/ODYSSEY B).

Further we will do a secondary analysis in all participants including the stratification information used in the main trial (ignoring weight band): antiretroviral therapy [ODYSSEY A/B]; Resistance Testing Availability [Yes/No]; and NRTI backbone for all participants. If insufficient patients are recruited <14kg in ODYSSEY B, provided results are similar across all patients and in ODYSSEY A with/without additional strata (stratum) for children <14kg, secondary analyses may be presented in preference to the primary.

4.8 Definition of New Resistance Mutations

Major NRTI, PI, NNRTI and INSTI resistance will be defined according to the most current IAS list of mutations at the time of analysis.

Activity of the background regimen will be defined according to the most current Stanford database algorithms at time of analysis.

4.9 Handling Adverse Events

Possible serious adverse events, notable events*, immune reconstitution inflammatory syndrome (IRIS) events, severe WHO 3 events, WHO 4 events and deaths occurring during the trial will be reviewed by an endpoint review committee blinded to treatment arm. Interim reports will differentiate between reviewed events and events awaiting review. Where an event has been reviewed, the review record will be used; otherwise the site report will be used.

Date event was diagnosed (with date from Form 30 for reviewed events and date of lab sample otherwise) will be used as the date of occurrence or date of worsening for adverse and staging events, unless specified otherwise below. An event which has a date of diagnosis or date of worsening before or on the date of randomisation will be considered to be pre-existing at randomisation and will be excluded from analysis.

All deaths that occur after the date of randomisation up to the trial censoring date will be included as ODYSSEY events. The "date of death" on Form 30 will be used as the date of occurrence for deaths.

SAEs will be analysed as episodes, with all components of the same clinical SAE presented as one episode (e.g. "Anaemia+renal failure"). Date of SAE will be defined as the date that the event met the seriousness criteria (i.e. date of death if resulted death, date of hospitalisation (from the site event Form 21) if resulted in hospitalisation). For SAEs meeting the seriousness criteria for reasons other than death or hospitalisation, the date the main event was diagnosed will be used.

Analyses of grade 3 or 4 AEs or adverse events leading to modification of ART/permanent discontinuation of study drugs will consider each component as separate events.

Grade 3 or 4 AEs, ART modifying events and notable events may be reported on more than one ERC form due to multiple SAEs (usually multiple hospital admissions) associated with the same underlying event. These can be identified by the same date of diagnosis and event code and the use of "OT" in the event description on second (and subsequent) ERC forms. All SAEs should be reported but the underlying event will only be counted once when reporting grade 3 or 4 AEs, ART-modifying events or notable events.

*notable events are pregnancy, suspected case of drug induced liver injury, ABC hypersensitivity reactions, suicidal ideation or behaviours, COVID-19 test positive and suspected or probable COVID-19 infection.

6. ANALYSIS PRINCIPLES

The two arms of the trial will be compared as randomised according to the intent-to-treat principle. Any participant randomised in a wrong stratum will be included in their incorrect stratum as randomised. Analyses of the main trial population will be performed across the whole trial population and for ODYSSEY A and B separately. Comparisons of endpoints will be undertaken adjusted for stratification factors (see 4.7) and adjusted for ODYSSEY A versus B.

Interactions between treatment arm and ODYSSEY A/ODYSSEY B strata will be tested and reported. Irrespective of the presence of an interaction effect, results will be presented in ODYSSEY A and B and in the total population for all outcomes.

Analyses of participants randomised <14kg and the whole trial population (< and ≥14kg) will follow the same principles, with adjustment for stratification factors as described under 4.7, and additional unadjusted analyses in participants <14kg. Bayesian estimation will be used in analysis of participants <14kg, incorporating evidence obtained from the ≥14kg participants as a prior distribution. Clinical opinion will be elicited on the relative weight to be allocated to the prior distribution. The Bayesian analysis will be reported alongside frequentist analyses of the <14kg participants and of the whole trial population (< and ≥14kg). If less than 80% of clinicians choose weights within a 30% absolute range, the Bayesian analysis will be reported as a secondary analysis and the frequentist analysis of the <14kg participants will be the primary analysis.

Analysis of the primary outcome is described under 6.6. Otherwise, descriptive statistics will be reported overall and by randomised group, and percentages will be of non-missing values, with the number (%) of non-missing values given if data are not complete. Statistical tests will be 2-sided and estimates will be presented with a 2-sided 95% CI. Appropriate transformations for all variables will be applied after inspection of the data.

Time-to-event outcomes will consider time from randomisation to the event date, using Kaplan-Meier estimation. Differences between groups in time-to-event outcomes will be tested using a adjusted Cox proportional hazard regression models. All hazard ratios or estimates of differences between groups will be presented with a 2-sided 95% confidence interval.

For the analysis of continuous variables, the following will be presented: by scheduled visits, the number of participants with a measurement and the mean and standard error. Changes from baseline in each randomised group at scheduled visits will be calculated using normal regression of absolute values (with 2-sided 95% confidence intervals), adjusting for the baseline measurement and stratification factors. Tests for the difference between randomised arms at each study visit will be presented. Average treatment differences through follow-up will be estimated with 2-sided 95% confidence intervals by fitting mixed linear models with random effects for intercept and fixed effects for treatment assignment and adjustment covariates. The mean (or median for skewed variables) change from baseline over time will also be plotted across scheduled visits by randomised group.

Differences between treatment groups in binary outcome variables within ODYSSEY A and B strata will be tested using Chi-squared tests or Fisher's exact tests as appropriate; differences between treatment groups within the total trial population will be tested using the Mantel-Haenszel Chi-squared test stratified by ODYSSEY A versus ODYSSEY B. Logistic regression models will be used for analyses adjusting for all stratification factors (presented with 2-sided 95% CI).

HIV-related events and AEs will be tabulated by event type and body system. The total number of events and the number of participants with at least one event will be given. Randomised groups will be compared in terms of time to first event. Event rates (per 100 person years) will be calculated as the number of events/total person years at risk*100 (presented with 2-sided 95%CI). Multiple events will be compared between randomised groups using Poisson regression models adjusted for clustering within individuals.

Percentages will be reported to 0 decimal places, unless <0.5% when they will be given to one decimal place. P-values will be given to 2 significant figures.

There will be no formal adjustment of α-values because of any interim analyses performed for IDMC meetings. Interim analysis will use early stopping guidelines and with the expected number of interim analyses, adjustment would make little difference in alpha.

7. ANALYSIS DETAILS

All descriptive and analytic statistics will be reported in the total population and separately for ODYSSEY A and B.

6.1 Enrolment and Eligibility

- Numbers screened, randomised, not randomised including tabulation of reason for non-randomisation
- Details of major and minor ineligibility and number excluded from analysis: number (%)
- Enrolment by arm and A/B strata: number (%)
- Enrolment by country and site: number (%)
- Enrolment by stratification factors: number (%)

6.2 Baseline Characteristics

Baseline characteristics will be summarised using descriptive statistics. No statistical tests will be used to compare treatment arms.

Demographics

- Sex: number (%), male, female
- Age at last birthday (years): number of patients, missing, mean, SD, median, IQR, range
- Age at last birthday (years)*: number(%), 0-<2/2-<6/6-<12/ 12-<18 years
- Weight (kg): number of patients, missing, mean, SD, median, IQR, range
- Weight (kg)*: number(%), 3-<6kg, 6-<10kg, 10-<14kg (for cohort <14kg), 14- <20kg, 20- <25kg, 25-<30kg, 30-<40kg, ≥40kg
- Height (cm): number of patients, missing, mean, SD, median, IQR, range
- BMI (kg/m^2): number of patients, missing, mean, SD, median, IQR, range
- Weight-for-age: median (IQR), range, number (%) in categories (<-3, -3 to <-2, -2 to <0, ≥0), missing
- Height-for-age: median (IQR), range, number (%) in categories (<-3, -3 to <-2, -2 to <0, ≥0), missing
- BMI-for-age: median (IQR), range, number (%) in categories (<-3, -3 to <-2, -2 to <0, ≥0), missing
- MUAC (cm): number of patients, missing, mean, SD, median, IQR, range
- Mode of infection: number (%), mother to child/blood product/sexual contact/ unknown/other
- Ethnic origin: number (%), white/black/mixed-black+white/asian/other
- EQ5D categories- mobility, self-care, usual activities, pain/discomfort, anxiety: number of patients, missing, median, IQR, range
- EQ5D Visual Scale: number of patients, missing, mean, SD, median, IQR

*more groupings may be added as required

HIV related parameters

- CD4%: number of patients, missing, mean, SD, median, IQR, range
- CD4%: number(%),<10%/10-<15%/15<20%/20-<25%/25-<30%/30-<40%/≥40%
- CD4 absolute: number of patients, missing, mean, SD, median, IQR, range
- CD4 absolute: number(%), <50/50-<100/100-<200/200-<350/350-<500/500-<1,000/1,000-<1,500/≥1,500
- CD8%: number of patients, missing, mean, SD, median, IQR, range
- CD8 absolute: number of patients, missing, mean, SD, median, IQR, range
- CD4/CD8 ratio: number of patients, missing, mean, SD, median, IQR, range
- Log_{10} Viral load: number of patients, missing, mean, SD, median, IQR, range
- Viral load: number (%), <400/400-<1,000/1,000-<10,000/10,000-<50,000/50,000-<100,000/100,000-<1,000,000/≥1,000,000
- History of WHO staging: number (%), 1/2/3/4
- Current or past WHO 3 or 4 events: number of events [number of children] categorised by event type

Pubertal status

- Tanner score, pubic hair: mean, SD, median, IQR, number (%): 1, 2, 3, 4, 5
- Tanner score, female breasts: mean, SD, median, IQR, number (%): 1, 2, 3, 4, 5
- Tanner score, male genitalia: mean, SD, median, IQR, number (%): 1, 2, 3, 4, 5

Laboratory data

- White cell count: number of patients, missing, mean, SD, median, IQR, range
- Haemoglobin: number of patients, missing, mean, SD, median, IQR, range
- MCV: number of patients, missing, mean, SD, median, IQR, range
- Platelets: number of patients, missing, mean, SD, median, IQR, range
- Neutrophils: number of patients, missing, mean, SD, median, IQR, range
- Lymphocytes: number of patients, missing, mean, SD, median, IQR, range
- Creatinine: number of patients, missing, mean, SD, median, IQR, range
- Total Bilirubin: number of patients, missing, mean, SD, median, IQR, range
- Aspartate transaminase number of patients, missing, mean, SD, median, IQR, range
- Alanine transaminase: number of patients, missing, mean, SD, median, IQR, range
- Alkaline phosphatase: number of patients, missing, mean, SD, median, IQR, range
- Calcium: number of patients, missing, mean, SD, median, IQR, range
- Phosphate: number of patients, missing, mean, SD, median, IQR, range
- Glucose: number of patients, missing, mean, SD, median, IQR, range
- Triglycerides: number of patients, missing, mean, SD, median, IQR, range
- LDL Cholesterol: number of patients, missing, mean, SD, median, IQR, range
- HDL Cholesterol: number of patients, missing, mean, SD, median, IQR, range
- Total Cholesterol: number of patients, missing, mean, SD, median, IQR, range
- Urine protein: number (%) negative, trace, +, ++, +++, ++++, missing
- Urine glucose: number (%) negative, trace, +, ++, +++, ++++, missing

Adherence [in ODYSSEY B Only]

- Adherence at baseline, as recorded on the Adherence Questionnaire Form, will be summarised using frequency tables and summary statistics.

Antiretroviral exposure [in ODYSSEY B Only]

- Number of different drugs ever received excluding in-utero/perinatal ART exposure: median [range], All classes/NRTI/PI/NNRTI /INSTI
- Number of ODYSSEY B participants exposed to ART classes: number (%), NRTIs+NNRTIs+PIs+INSTIs/ NRTIs+NNRTIs+INSTIs only/ NRTIs+NNRTIs+PIs only /NRTIs + NNRTIs only/ NRTIs + PIs only/ NRTIs + INSTIs only/ NRTIs /monotherapy
- Cumulative ART exposure (years): median [range], All classes/NRTI/PI/NNRTI
- ART regimen prior to randomisation: number (%), NNRTI + NRTIs, bPI +NRTIs, regimen combinations >10% will also be described

6.3 Follow-Up

- Description of follow-up to trial censoring date: distribution of time from randomisation to latest follow up (defined as date last known to be alive) in weeks, median (IQR), range
- Number of patients who have died and who are confirmed as having withdrawn consent. At interim analyses, number (%) of patients last seen in 0-12 weeks, 12-24 weeks, 24-36 weeks, 36-48 weeks and >48 weeks prior to data freeze date, number potentially lost to follow-up (either confirmed as withdrawn or not seen for >24 weeks). At end of trial, number (%) of patients who did not have a face-to-face visit on/after 24th April 2020 visit; number (%) of patients who did not have a face-to-face visit on/after 24th April 2020 and primary endpoint status is unknown, number (%) of patients (who did not have a face-to-face visit on/after 24th April 2020) seen in 0-12 weeks, 12-24 weeks, 24-36 weeks, 36-48 weeks and >48 weeks prior to trial censoring date. Similar data will be presented for children recruited <14kg using a trial censoring date of 28 June 2021.
- Summary of attendance at the end of randomised phase visit
- Number (percentage) of scheduled study visits attended, overall and by assessment week

6.4 Antiretroviral Therapy Received

- ART prescribed at randomisation: distribution of time from randomisation to prescription in days, same day, 1-3, 4-14, $\geq$14, range; reasons for gap of $\geq$14 days
- Initial ODYSSEY regimen: number (%) by NRTI backbone and third agent component of regimen. Number (%) not starting ART, starting non-protocolled ART, starting allocated regimen

- Tabulation of substitutions and changes to ART regimen: number (%) on strict initial regimen, initial regimen with substitutions, median number of substitutions, IQR, % of follow-up time spent on strict initial or initial regimen with substitution. Number (%) changed from initial regimen: for toxicity, for treatment failure, for pregnancy.
- List of substitutions and changes in initial treatment regimen: Trial No., A/B Strata, Arm, Week, regimen before, regimen after, reason for substitution or change
- Time from randomisation to change in third agent for toxicity, failure or pregnancy will be assessed using a log-rank test and Cox proportional hazard regression models (adjusted for the stratification factors). A hazard ratio will be presented with a 2-sided 95% confidence interval. This analysis will be done provided there are >15 events in total.

6.5 Availability of HIV-1 RNA Measurements

- Tabulation of available HIV-1 RNA measurement by visit week: number (%), available/missing.

6.6 Primary Efficacy Outcome

The primary outcome will be assessed by time to clinical or virological failure (section 2.1) (ODYSSEY A and B combined) using Kaplan Meier curves to estimate the proportion of children failing in each arm at any time up to the week 96 censoring date (96 weeks from date of randomisation). The primary analysis will adjust for stratification factors as described under 4.7. Secondary analyses will be done adjusting for ODYSSEY A versus B strata only. The survival curve for each combination of strata and randomised group will be calculated using a Cox model adjusting for stratification factors and randomised group. The average survival curve for each randomised group will be estimated as a weighted average of the corresponding stratum-specific survival curves, with weights proportional to the number of individuals in each stratum across both randomised groups at baseline. The mean of these differences at week 96 is the point estimate for the difference in overall survival function between DTG and SOC arm. Two-sided 95% confidence intervals for the proportions failing by treatment arm will be estimated by bias corrected bootstrap. A two sided bias-corrected 95% CI for the difference in proportion (DTG – SOC) will be calculated with bootstrap standard errors. The bootstrapping will sample 1000 times and be stratified by stratification factors.

If the upper limit of the confidence interval is <10% DTG+2NRTIs will be considered non-inferior to SOC.

Since only 85 children were recruited weighing <14kg, confidence intervals for the proportions failing by treatment arm will be estimated by bootstrap on the log(-log(S(t)) scale, assuming Normal theory. The confidence interval for the risk difference will be estimated using bias-corrected bootstrap.

Secondary analyses will summarise the rate of the composite primary endpoint per 100 person years by randomised group, a treatment HR (DTG-regimen relative to SOC-regimen) estimated by a Cox proportional hazards regression model.

i. Sensitivity analyses for the primary endpoint

The following sensitivity analyses will be performed:

i. Restricted to the per protocol population.
ii. Excluding participants in the SOC arm who initiated ODYSSEY treatment on an integrase inhibitor.
iii. Restricted to the per protocol population (as in (i)) and excluding participants in the SOC arm who initiated ODYSSEY treatment on an integrase inhibitor (as in (ii)).
iv. Censoring participants who have one unconfirmed HIV RNA ≥ 400 c/ml, with a treatment switch for failure before a confirmatory HIV RNA ≥ 400 c/ml, at the first HIV RNA ≥ 400 c/ml; and censoring participants who switched treatment for failure without reaching the primary endpoint, at time of switch.
v. Using actual dates of confirmatory viral loads (or switch for treatment failure) for date of failure.
vi. Using the date of the first VL ≥400 c/ml at or after 36 weeks for date of failure.
vii. Adjusting for calendar time corresponding to any major changes in eligibility criteria (recruitment to younger age-groups/lower weight bands).

ii. Subgroup analyses for the primary endpoint

Subgroup analyses will be performed to assess heterogeneity in differences between randomised groups according to:

- Sex
- Age groups
- Weight bands
- Baseline HIV RNA
- Baseline CD4 percent
- Routine availability at site of resistance tests for children failing treatment
- NRTI backbone
- bPI vs non-bPI SOC regimens (if sufficient data)

6.7 Secondary Efficacy Analyses

Difference in proportion with clinical or virological failure (as defined using primary outcome definition) by 48 weeks

The difference in the proportion of participants with clinical or virological failure by 48 weeks will be analysed in the same way as by 96 weeks. See 'Primary Efficacy Analysis' for further details.

Proportion of children with VL <50 c/ml at 48 and 96 weeks
Proportion of children with VL <400 c/ml at 48 and 96 weeks

Participants with VL<50 (or <400) c/ml at 48 and 96 weeks will be compared between arms based on (i) crude proportions and (ii) using the FDA snapshot algorithm[2]. The estimated difference in the proportion of suppressed patients at weeks 48 and 96 weeks will be provided with 95% confidence interval; in the total population this will be estimated by the Mantel-Haenszel weighted mean of proportions in ODYSSEY A and B. The Chi-squared test, Fisher's exact test or Mantel-Haenszel Chi-squared test will be use to assess differences between arms. Logistic regression will be used for analyses adjusting for all stratification factors.

Rate of clinical events (WHO 4, severe WHO 3 events and death) over 96 weeks

The number of clinical events occurring during follow-up will then be tabulated by number (%) of events/people for each clinical event separately and for one or more event. The number of children with at least one event will be compared between arms using the Chi-squared test, Fisher's exact test or Mantel-Haenszel Chi-squared test (as appropriate). Logistic regression will be used for analyses adjusting for all stratification factors. Event rates will be calculated as the number of events/total person years at risk*100 and will be reported per 100 person years including 95% CI. Poisson regression will be used to calculate the incidence rate ratios of clinical events in the DTG arm versus SOC arm adjusted for clustering of events by individuals. The rate ratio will be given along with the 95% CI and p-value. Hazard ratios for first events will also be calculated using Cox proportional hazard regression models.

Time to any new or recurrent AIDS defining event (WHO 4) or severe WHO 3 events confirmed by Endpoint Review Committee

Follow-up time will be summarised and number (%) of first events will be tabulated for WHO 4 and severe WHO 3 events. Randomised groups will be compared in terms of time to first event using a log-rank test and Cox proportional hazard regression models (stratified by the stratification factors). A hazard ratio will be presented with a 2-sided 95% confidence interval. Event rates will be calculated as the number of first events/total person years at risk*100 and will be reported per 100 person years including 95% confidence intervals.

Change in CD4 count and percentage and CD4/CD8 ratio from baseline to weeks 48 and 96

Changes in CD4 (absolute and percentage) and CD4/CD8 ratio from baseline will be reported by scheduled visit week. The overall baseline mean will be calculated using all those with a CD4% or CD4 absolute measurement at baseline. Change from baseline to each of the scheduled follow-up visit weeks will be calculated using normal regression adjusting for baseline and ODYSSEY A versus ODYSSEY B. An additional adjusted analysis will be performed adjusting for baseline and stratification factors.

For each of the scheduled follow-up visit weeks the following will be reported:

- Change from baseline, by arm: mean (SE)
- Unadjusted (i.e. only adjusting for baseline and A/B strata) difference between the two arms: mean (SE), p-value.
- Adjusted (i.e. adjusting for baseline and stratification factors) difference between the two arms: mean (SE), 95% CI, p-value.

For each analysis, a graph showing the change from baseline by week including 95% CI will be presented.

Average treatment differences through follow-up will be estimated with 2-sided 95% confidence intervals by fitting linear mixed models with random effect for intercept and fixed effects for treatment assignment, post-randomisation study visits and adjustment covariates (baseline level of the outcome variable and the stratification factors used in the randomisation).

Proportion developing new resistance mutations (see Section 4.8 for definitions)

The number and % of participants with new resistance mutations by type of mutation will be described in participants who have reached a virological component of the primary endpoint. The proportion of participants developing new resistance mutations will be compared between arms using the Chi-squared test, Fisher's exact test or Mantel-Haenszel Chi-squared test, as appropriate. Logistic regression will be used for analyses adjusting for all stratification factors. 95% confidence interval for the difference in proportion will be provided.

A walk back approach will be used to describe development of resistance from baseline.

6.8 Secondary Safety Outcomes

The following safety outcomes will be examined:

Change in total cholesterol, triglycerides and lipid fractions (LDL, HDL, total cholesterol/HDL ratio, non-HDL) from baseline to weeks 48 and 96

For each measurement, changes from baseline will be reported by scheduled visit week. The overall baseline mean will be calculated using all those with a measurement at baseline. Change from baseline to each of the scheduled follow-up visit weeks will be calculated using normal regression adjusting for baseline and ODYSSEY A versus ODYSSEY B. An additional adjusted analysis will be performed adjusting for baseline and stratification factors.

For each of the scheduled follow-up visit weeks the following will be reported:

- Change from baseline, by arm: mean (SE)
- Unadjusted (i.e. only adjusting for baseline and A/B strata) difference between the two arms: mean (SE), p-value.
- Adjusted (i.e. adjusting for baseline and stratification factors) difference between the two arms: mean (SE), 95% CI, p-value.

For each analysis, a graph showing the change from baseline by week including 95% CI will be presented.

Average treatment differences through follow-up will be estimated with 2-sided 95% confidence intervals by fitting linear mixed models with random effect for intercept and fixed effects for treatment assignment, post-randomisation study visits and adjustment covariates (baseline level of the outcome variable and the stratification factors used in the randomisation).

The secondary endpoints of change in total cholesterol, triglycerides and lipid fractions (LDL, HDL) will be assessed for superiority of DTG compared to SOC. Superiority will be regarded as proven if the mean adjusted change in total cholesterol at 96 weeks is lower in the DTG arm and the P value is <0.05.

Incidence of serious adverse events
Incidence of new clinical and laboratory grade 3 and 4 adverse events
Incidence of adverse events (of any grade) leading to treatment modification

The number (%) of events/people will be tabulated for each clinical event separately and for one or more event. The number of children with at least one event will be compared between arms using the Chi-squared test, Fisher's exact test or Mantel-Haenszel Chi-squared test (as appropriate). Logistic regression will be used for analyses adjusting for all stratification factors. Event rates will be calculated as the number of events/total person years at risk*100 and will be reported per 100 person years including 95% CI. Poisson regression will be used to calculate the incidence rate ratios of clinical events in the DTG arm versus SOC arm adjusted for clustering of events by individuals. The rate ratio will be given along with the 95% CI and p-value. Hazard ratios for first events will also be calculated using Cox proportional hazard regression models.

6.9 Other Secondary Outcomes

Quality of life
A descriptive analysis of each dimension of the EQ5D will also be undertaken at each study visit.

Adherence and acceptability
Carer completed and patient completed adherence and acceptability questionnaires will be analysed separately. A descriptive analysis of adherence questionnaire will be done. The proportion of questionnaires where the participant/carer reports missing a dose within the last week will be compared between randomised groups.

6.10 Other Outcomes

Change in biochemistry and haematology measures
Analysis of the change in biochemistry and haematology measures will be undertaken in the same way as changes in metabolic parameters; please see "Change in total cholesterol, triglycerides and lipid fractions (LDL, HDL) from baseline to weeks 48 and 96".

Change in growth measures
Analysis of the change in height, weight, body mass index (BMI), and mid upper arm circumference (MUAC) will be undertaken in the same way as changes in metabolic parameters; please see "Change in total cholesterol, triglycerides and lipid fractions (LDL, HDL) from baseline to weeks 48 and 96".

Incidence of notable events* or immune reconstitution inflammatory syndrome (IRIS), examined separately.
Analysis of the incidence of notable events or IRIS will be undertaken in the same way as other events; please see "Incidence of serious adverse events". We will also examine incidence of IRIS in a subgroup analysis by baseline CD4 count (≥ vs. < 200 cells per µL).

*notable events are pregnancy, suspected case of drug induced liver injury, ABC hypersensitivity reactions, suicidal ideation or behaviours, COVID-19 test positive and suspected or probable COVID-19 infection.

Incidence of newly diagnosed or worsening lipodystrophy
Follow-up time will be summarised and number (%) of first events will be tabulated. Randomised groups will be compared in terms of time to first event using a log-rank test and Cox proportional hazard regression models (stratified by the stratification factors). A hazard ratio will be presented with a 2-sided 95% confidence interval. Event rates will be calculated as the number of first events/total person years at risk*100 and will be reported per 100 person years including 95% confidence intervals.

Change in sleep and mood questionnaire
Carer completed and patient completed mood and sleep questionnaires will be analysed separately. For mood symptoms, the proportion of questionnaires where the participant or carer reports a symptom will be compared between arms over time using logistic mixed models with random effect for intercept and fixed effects for treatment assignment, post-randomisation study visits and adjustment covariates if any (total adjusted for A and B stratification, no adjustment in A and B). For sleep symptoms, ordinal logistic mixed models will be used to compare outcomes across treatment arms over time with a random effect for intercept and fixed effects for treatment arm, study visits and adjustment covariates if any (total adjusted for A and B stratification, no adjustment in A and B). Analyses will

not be adjusted for baseline levels as questionnaires were implemented after the trial had starting and a baseline questionnaire is missing in many participants.

Descriptive outcomes in participants switching to DTG as third-line
Viral loads and clinical events in participants who experience treatment failure on second-line ART and subsequently changed to dolutegravir-based regimen for third-line will be described.

6.11 Testing multiple outcomes

We will make no adjustment to p-values or confidence intervals to allow for testing multiple secondary outcomes. The ODYSSEY primary outcome is a composite outcome of virological failure, clinical events (WHO 4 and severe WHO 3 events) and death. The primary analysis is non-inferiority of DTG versus SOC. If the 95% CI for the treatment effect (difference in proportion failing by 96 weeks (DTG-SOC)) lies below the non-inferiority margin, then we will also test for superiority; because this is a closed test procedure there is no issue of multiplicity.

Secondary outcomes are divided in the protocol into efficacy outcomes, safety outcomes and other patient-reported outcomes. For safety outcomes it is appropriate to test each independently since it is important to identify any risks associated with DTG. Change in total cholesterol from baseline to week 96 has been defined as the primary safety outcome for the assessment of superiority of DTG versus SOC.

The secondary efficacy outcomes are mostly components of the primary outcome or very closely related to the primary outcome (virological and clinical outcomes). We will not adjust for multiple testing for these since they are correlated with the primary outcome (so standard adjustments are conservative). Resistance and immunological outcomes are considered exploratory and significance tests on these outcomes alone will not be used to conclude superiority.

We will report significance tests for differences between treatment arms for patient-reported outcomes, but if we have failed to demonstrate non-inferiority of DTG versus SOC for the primary outcome, we will not use significance tests for these patient-reported outcomes to conclude superiority.

8. SUBGROUP ANALYSES

7.1 Analyses by Dose and Weight-band

Safety, tolerability and efficacy outcomes will also be examined by time-updated DTG dose and weight band.

9. ANCILLARY STUDIES AND SUBSTUDIES

The following substudies will be conducted in subsets of participants:

- Pharmacokinetic substudy to examine DTG dosing in children <40kg
- (Lower WB-PK, WB-PK1 and WB-PK2)Pharmacokinetic substudy in participants developing TB (TB-PK)
- Immunology/virology substudy
- Qualitative substudy
- Youth Trial Board Project

Further details on these ancillary studies and substudies are given in the appendices of the trial protocol. Analysis plans will be developed in separate documents.

PK and accompanying safety data on PK participants in the DTG arm are being analysed to support the ViiV submission for FDA and EMA licensing of DTG dosing in children <40kg. A separate RAP (reporting and analysis plan) will be prepared.

REFERENCES

1. Parienti J-J, Verdon R, Massari V. Methodological standards in non-inferiority AIDS trials: moving from adherence to compliance. *BMC Medical Research Methodology.* 2006;6(1):46.
2. Food Drug Administration Center for Drugs Evaluation Research. Human Immunodeficiency Virus-1 Infection: Developing Antiretroviral Drugs for Treatment - Guidance for Industry. In. Maryland, USA: FDA; 2015.

APPENDIX I

Table 1. ADAPTED WHO CLINICAL STAGING OF HIV/AIDS FOR PATIENTS WITH CONFIRMED HIV INFECTION

**Children (<15 years of age)**	**Adolescents (≥15 years of age)**
**Clinical Stage 3 (*= severe WHO clinical stage 3 for the ODYSSEY trial)**	
• Unexplained moderate malnutrition or wasting not adequately responding to standard therapy*<br>• Unexplained persistent diarrhoea (14 days or more)<br>• Unexplained persistent fever (above 37.5°C intermittent or constant, for longer than one month)<br>• Persistent oral candidiasis (after first 6–8 weeks of life)*<br>• Oral hairy leukoplakia<br>• Acute necrotizing ulcerative gingivitis or periodontitis<br>• Lymph node tuberculosis<br>• Pulmonary tuberculosis<br>• Severe recurrent bacterial pneumonia<br>• Symptomatic lymphoid interstitial pneumonitis*<br>• Chronic HIV-associated lung disease including bronchiectasis*<br>• Unexplained anaemia (<8 g/dl), neutropaenia (<0.5 × 10^9 per litre)and or chronic thrombocytopaenia (<50 × 10^9 per litre)	• Unexplained severe weight loss (>10% of presumed or measured body weight)*<br>• Unexplained chronic diarrhoea for longer than one month<br>• Unexplained persistent fever (above 37.6°C intermittent or constant, for longer than one month)<br>• Persistent oral candidiasis*<br>• Oral hairy leukoplakia<br>• Pulmonary tuberculosis (current)<br>• Severe bacterial infections (such as pneumonia, empyema, pyomyositis, bone or joint infection, meningitis or bacteraemia)<br>• Acute necrotizing ulcerative stomatitis, gingivitis or periodontitis<br>• Unexplained anaemia (<8 g/dl), neutropaenia (<0.5 × 10^9 per litre) or chronic thrombocytopaenia (<50 × 10^9 per litre)
**Clinical Stage 4**	
• Unexplained severe wasting, stunting or severe malnutrition not responding to standard therapy<br>• Pneumocystis pneumonia<br>• Recurrent severe bacterial infections (such as empyema, pyomyositis, bone or joint infection or meningitis but excluding pneumonia)<br>• Chronic herpes simplex infection (orolabial or cutaneous of more than one month's duration or visceral at any site)<br>• Oesophageal candidiasis (or candidiasis of trachea, bronchi or lungs)<br>• Extrapulmonary tuberculosis<br>• Kaposi's sarcoma<br>• Cytomegalovirus infection: retinitis or cytomegalovirus infection affecting another organ, with onset at age older than one month<br>• Central nervous system toxoplasmosis (after one month of life)<br>• Extrapulmonary cryptococcosis (including meningitis)<br>• HIV encephalopathy<br>• Disseminated endemic mycosis (coccidiomycosis or histoplasmosis)<br>• Disseminated non-tuberculous mycobacterial infection<br>• Chronic cryptosporidiosis (with diarrhoea)<br>• Chronic isosporiasis<br>• Cerebral or B-cell non-Hodgkin lymphoma<br>• Progressive multifocal leukoencephalopathy<br>• Symptomatic HIV-associated nephropathy or HIV-associated cardiomyopathy	• HIV wasting syndrome<br>• Pneumocystis pneumonia<br>• Recurrent severe bacterial pneumonia<br>• Chronic herpes simplex infection (orolabial, genital or anorectal of more than one month's duration or visceral at any site)<br>• Oesophageal candidiasis (or candidiasis of trachea, bronchi or lungs)<br>• Extrapulmonary tuberculosis<br>• Kaposi's sarcoma<br>• Cytomegalovirus infection (retinitis or infection of other organs)<br>• Central nervous system toxoplasmosis<br>• HIV encephalopathy<br>• Extrapulmonary cryptococcosis including meningitis<br>• Disseminated non-tuberculous mycobacterial infection<br>• Progressive multifocal leukoencephalopathy<br>• Chronic cryptosporidiosis (with diarrhoea)<br>• Chronic isosporiasis<br>• Disseminated mycosis (coccidiomycosis or histoplasmosis)<br>• Recurrent non-typhoidal Salmonella bacteraemia<br>• Lymphoma (cerebral or B-cell non-Hodgkin) or other solid HIV-associated tumours<br>• Invasive cervical carcinoma<br>• Atypical disseminated leishmaniasis<br>• Symptomatic HIV-associated nephropathy or symptomatic HIV-associated cardiomyopathy
